# TRIM14 is a Putative Tumor Suppressor and Regulator of Innate Immune Response in Non-Small Cell Lung Cancer

**DOI:** 10.1038/srep39692

**Published:** 2017-01-06

**Authors:** Josephine Hai, Chang-Qi Zhu, Tao Wang, Shawna L. Organ, Frances A. Shepherd, Ming-Sound Tsao

**Affiliations:** 1 Princess Margaret Cancer Centre, University Health Network, Toronto, ON, Canada; 2 Department of Medical Biophysics, University of Toronto, Toronto, ON, Canada; 3 Department of Medical Oncology, Dana-Farber Cancer Institute, Boston, MA, USA; 4 Department of Medicine, Harvard Medical School, Boston, MA, USA

## Abstract

Non-small-cell lung carcinoma (NSCLC) accounts for 85% of malignant lung tumors and is the leading cause of cancer deaths. Our group previously identified Tripartite Motif 14 (*TRIM14*) as a component of a prognostic multigene expression signature for NSCLC. Little is known about the function of TRIM14 protein in normal or disease states. We investigated the functional and prognostic role of *TRIM14* in NSCLC using *in vitro* and *in vivo* perturbation model systems. Firstly, a pooled RNAi screen identified TRIM14 to effect cell proliferation/survival in NSCLC cells. Secondly, silencing of TRIM14 expression significantly enhanced tumor growth in NSCLC xenograft mouse models, while exogenous TRIM14 expression attenuated tumorigenesis. In addition, differences in apoptotic activity between TRIM14-deficient and control tumors suggests that TRIM14 tumor suppressor activity may depend on cell death signaling pathways. TRIM14-deficient cell lines showed both resistance to hypoxia-induced cell death and attenuation of interferon response via STAT1 signaling. Consistent with these phenotypes, multivariate analyses on published mRNA expression datasets of over 600 primary NSCLCs demonstrated that low *TRIM14* mRNA levels are significantly associated with poorer prognosis in early stage NSCLC patients. Our functional data therefore establish a novel tumor suppressive role for TRIM14 in NSCLC progression.

Lung cancer is the leading cause of cancer deaths worldwide and non-small cell lung cancer (NSCLC) accounts for roughly 80% of those cases^1^,^2^. Although many tumor suppressor genes and oncogenes pertinent to NSCLC oncogenesis have been characterized in the last two decades, the overall survival rate for NSCLC patients remains at 16% due to late stage diagnosis and unsuccessful treatments. The low efficacy of current diagnostic and treatment strategies underscores the importance of identifying novel mechanisms regulating NSCLC progression as new potential prognostic markers and therapeutic targets in NSCLC.

The tripartite motif (TRIM) family proteins are defined by a conserved domain architecture composed of three zinc-binding regions: a RING finger, one or two B-boxes, and a coiled-coil domain^3^. Originally known as KIAA0129, TRIM14 was first discovered as overexpressed in HIV-infected human and simian lymphomas by subtractive hybridization^4^,^5^. To date, very little is known about the biological and molecular mechanisms mediated by TRIM14 in either normal or pathogenic states. Initial studies on the mouse homolog of TRIM14, *Pub*, revealed that the protein’s B- box zinc finger domain inhibits the activity of PU.1, an SP1 transcription factor mouse homolog with a role in hematopoiesis^6^. Zhou *et al*.^7^ demonstrated that TRIM14 mediates immune responses against viral infection by recruiting NF-kB essential modulator (NEMO) to the mitochondrial antiviral signaling (MAVs) adaptor complex to activate the interferon (IFN) regulatory factor 3. Nenasheva *et al*.^8^ found that overexpression of TRIM14 in human HEK293T cells resulted in the transcriptional upregulation of many genes involved in innate immune response. Most recently, Su *et al*.^9^ showed that TRIM14 promoted cell proliferation via NF-κB activation both *in vitro* and *in vivo*. Collectively, emerging evidence suggests that aberrant TRIM14 expression is associated with mechanisms regulating a complex interplay between immune response and cancer. Here, we have combined a genetic screen, clinical data, functional assays and *in vivo* xenograft models to provide strong evidence that TRIM14 plays a novel tumor-suppressive role in lung cancer.

## Materials and Methods

### *In silico* prognostic evaluation of *TRIM14* expression

To assess the prognostic value of *TRIM14* expression, *in silico* analyses were performed on published microarray data from four patient cohorts. JBR.10 was a phase 3 randomized trial of adjuvant chemotherapy (cisplatin and vinorelbine) *versus* observation in stage IB-II patients. The prognostic value of *TRIM14* was assessed in the expression data of 62 patients in the observation arm^10^,^11^. The National Cancer Institute Directors’ Challenge Consortium (DCC) cohort included 442 adenocarcinoma patients from 4 North American cancer centers. Excluding patients from the JBR.10 cohort contributed into this DCC study and patients who received adjuvant chemo/radiotherapy, expression data from the 311 patients were used for prognostic analysis^12^. The University of Michigan cohort consisted of 129 stage I-III squamous cell carcinomas^13^. The University Health Network cohort consisted of 181 stage I-II NSCLCs^14^. Gene expression analyses from the above 3 cohorts were performed using the Affymetrix U133A microarray. The association of the expression of *TRIM14* and survival was evaluated using Cox proportional hazards regression in SAS v9.2 (SAS Institute) with gene expression as a continuous variable. Datasets in this publication are accessible through the National Center for Biotechnology Information Gene Expression Omnibus (http://www.ncbi.nlm.nih.gov/geo/) through GEO Series accession number GSE68465, GSE4573 and GSE14814, respectively.

### Cell culture

Human NSCLC cell lines NCI-H1650, H520, H157, H358, H3255 and H1395 were obtained from the American Type Culture Collection (ATCC; Manassas, VA) and cultured in RPMI-1650 media supplemented with 10% Fetal Bovine Serum (FBS; Hyclone Europe, Ltd., Cramlington, UK) and antibiotics. Human embryonic kidney 293T (HEK293T) cells were cultured in DMEM media supplemented with 10% FBS and antibiotics. All cells were cultivated at 37 °C and 5% CO_2_. Authentication of human cell lines was done by short tandem repeat (STR) DNA profiling analysis (Supplemental Table 6). For anoxic treatment, cells were cultured in HypOxygen H85 workstation (Don Whitley Scientific) and the chamber atmosphere consisted of 5% H_2_, 5% CO_2_, <0.02% O_2_ and 90% N_2_.

### Lentiviral shRNA screen and stable isogenic cell line generation

Each gene was targeted by four or five constructs obtained from the RNAi Consortium (TRC; Toronto, ON). Lentiviral shRNA expression vectors (pLKO.1 backbone) were transfected into 293T cells in culture plates using protocols from TRC (http://portals.broadinstitute.org/gpp/public/). Targets cells were infected with lentivirus at 0.4 multiplicity of infection according to TRC protocols. The medium containing 2 μg/ml puromycin was added 24 hours post transfection to select for cells stably transduced with short hairpin RNA (shRNA).

shRNA against human *TRIM14* used for further experiments included: shTRIM14.A (TRCN0000061828), shTRIM14.B (TRCN0000061832), and non-specific control shGFP (TRCN0000072179). Human full length TRIM14 cDNA plasmid, pOTB7-TRIM14, was obtained commercially (4299815; Fisher Scientific, Waltham, MA) and was subcloned into our modified Gateway recombination lentiviral expression vector, pLKO.puro.DEST^15^, containing a puromycin selection marker. All vectors were sequence confirmed.

Transient transfections and virus preparation in HEK293T cells were performed using Fugene reagents (Promega, Madison, WI) as per manufacturer’s protocol. Lentiviruses were prepared by transfecting three packaging plasmids into 293T cells using protocols from TRC (Broad Institute). Stable cell lines were isolated following viral transduction and selection with puromycin antibiotics (1 μg/mL).

### Western blot analysis, antibodies and immunohistochemistry

Whole cell extracts were lysed with lysis buffer (10 mM Tris [pH 8.0], 1% NP-40, 2 mM EDTA, 150 mM, 0.1 mM Na_3_VO_4_ and protease inhibitors [Roche]), resolved by SDS-PAGE and transferred to polyvinyliden fluoride membranes. Primary antibodies included: anti-TRIM14 (Proteintech), FLAG M2 affinity gel, Protein G-agarose beads, β-actin (Sigma), AIF (Invitrogen), p62, HAX1 (Genetex), phosphor-STAT1 (Cell Signaling), STAT1 (Invitrogen). After blocking, membranes were incubated with relevant antibodies and probed with corresponding HRP-conjugated secondary antibodies (Cell Signaling). All films were developed with ECL-Plus reagents (GE healthcare, Piscataway, NJ).

Formalin-fixed paraffin-embedded tissues were cut at 4-μm thickness and dried in a 60 °C oven overnight. Immunohistochemistry was performed using the peroxidase anti-peroxidase technique following a microwave antigen retrieval procedure. Ki67 and cleaved caspase-3 antibodies were used at 1:500 dilution following pepsin digestion. Whole slides were scanned using the Aperio Scanscope XT (Vista, CA). Percentage of cells with positive Ki67 or cleaved caspase-3 staining were independently blind scored (TW and JH).

### Immunoprecipitation for liquid chromatography-tandem mass spectrometry (LC-MS/MS)

Forty-eight hours after transfection, cells were lysed and equal amounts of extract were precleared using Protein G-agarose beads (Sigma). Extracts were incubated separately with anti-FLAG affinity gel overnight at 4 °C. After washing the immunoprecipitates, bound protein complexes were eluted using 0.15% trifluoroacetic acid (TFA), reduced using 45 mM dithiothreitol and incubated with 1 μg TPCK trypsin overnight. Trypsinized peptides were acidified with 20% TFA, purified using C-18 spin columns (Pierce, IL, USA) and vacuum concentrated. The samples were loaded onto a 75 μm ID X 50 cm (2 μm C18) analytical column (EASY-Spray, Thermo-Fisher Scientific, Odense Denmark). The peptides were eluted over 2 hours at 250 nl/minute using a 0 to 35% acetonitrile gradient in 0.1% formic acid using an EASY nLC 1000 nano-chromatography pump (Thermo-Fisher Scientific, Odense Denmark). The peptides were eluted into a LTQ Velos-Orbitrap Elite hybrid mass spectrometer (Thermo-Fisher, Bremen, Germany) operated in a data dependant mode. MS was acquired at 240,000 FWHM resolution in the FTMS (using a target value of 5 × 10^5^ ions) and MS/MS was carried out in the linear ion trap. 10 MS/MS scans were obtained per MS cycle using a target of 1 × 10^4^ ions and a maximum injection time of 50 ms, and all ions passing the monoisotopic precursor selection (MIPS) filter were fragmented.

### MTS cell proliferation, colony formation and apoptosis assays

Cell proliferation/viability was evaluated by the tetrazolium dye (MTS) assay (Promega, Madison, WI). Each cell line was plated at a seeding density to give logarithmic growth over the course of the assay in a 96-well tissue culture plate. The concentration of final product is measured by absorbance at 490 nm and is proportional to the viable cell number in each well. For soft agar assays, 5 × 10^3^ to 10^4^ cells were suspended in 0.8% low melting point agarose (Difco Laboratories Inc., Detroit, MI) at room temperature, mixed with an equal volume of 2x concentrated culture media, and plated onto an agarose bed consisting of 2% low melting point agarose and the same medium. After 3–6 weeks, colonies were stained with neutral red and were enumerated using a Leica MZ FLIII Stereomicroscope (Leica, Germany).

Apoptosis was determined using the Annexin V assay (Biolegend, San Diego, CA) and cell cycle was analyzed using the Propidium Iodide assay (Invitrogen, MA). After treatment, cells were collected and stained with Annexin V-FITC and/or propidium iodide solution as per the manufacturer’s protocol and analyzed by flow cytometry. Cell death was recorded in a FACSCanto II (BD Biosciences, Mississauga, ON) in the total population (10,000 cells) and data were analyzed using FACSCanto II software and ModFit Software (Verity Software House, Topsham, ME).

### Animals and subcutaneous tumorigenicity assays

Severe combined immunodeficient (*SCID*) and Non-obese diabetic severe combined immunodeficient (*NOD-SCID*) mice were bred on site and obtained from the Ontario Cancer Institute (OCI; Toronto, ON) animal facility. All manipulations were performed under sterile conditions in a laminar flow hood, in accordance with procedures approved by the OCI Animal Care Committee. H1650 (3 × 10^6^) and H157 (2 × 10^6^) cells were injected subcutaneously in the right flank of 6-week-old *SCID* mice (*n* = 7–10 per group). Two million cells of H3255 cells were injected into 6-week-old *NOD-SCID* mice. Mice were examined every 2–3 days, and tumor length and width were measured using calipers. Tumor volume was calculated using the following formula: (length × width^2^)π/6. Mice were sacrificed once the humane endpoint (~1.5 cm diameter) was reached. At sacrifice, portions of tumors were snap-frozen and stored in liquid nitrogen or were fixed in 10% buffered formalin for routine histopathologic processing.

### *In vivo* ubiquitylation assays

Forty-eight hours post transfection, HEK293T cells expressing HA-Ub and 3xFLAG-TRIM14 were treated with 10 μM of MG132 for 4 hours. After treatment, the cells were washed twice and lysed with lysis buffer containing 2 mM N-ethylmaleimide, which blocks most deubiquitylating enzyme activity. Extracts were denatured by adding 1% SDS followed by boiling for 10 min. Lysates were then quenched with lysis buffer containing 1% Triton X-100 and incubated on ice for 30 min. Cleared lysates were quantified and an equal amount of each lysate was used for immunoprecipitation with anti-FLAG M2 affinity gel. Beads were washed with lysis buffer three times and boiled in SDS-PAGE sample buffer with β-mercaptoethanol for 5 min before separation on a SDS-PAGE gel.

### Statistics

All data are presented as mean ± SEM. Statistical significance was determined using two-tailed Student’s t test and mixed-model ANOVA for colony formation and MTS assay, respectively. Difference in tumor growth rates of xenografts was tested using mixed-model ANOVA. Tests that produced *P* ≤ 0.05 were considered to be significant. All statistical analyses were performed with GraphPad Prism 6.0 (La Jolla, CA).

### Ethics approval

All animal experiments were performed in accordance with procedures approved by the Ontario Cancer Institute (Toronto, ON) Animal Care Committee.

## Results

### TRIM14 negatively affects proliferation/survival of NSCLC cell lines *in vitro*

**Figure 1:**
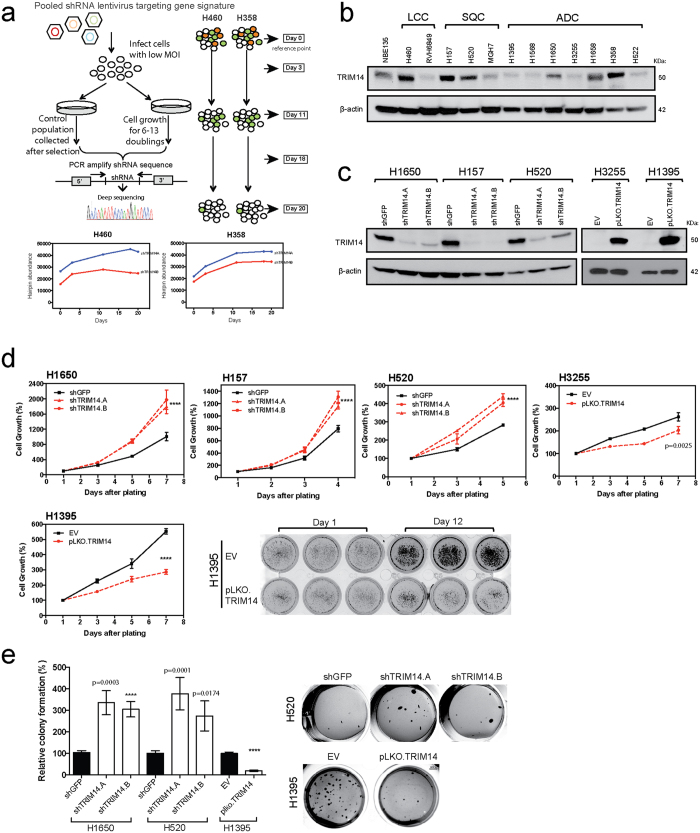
TRIM14 negatively affects proliferation/survival of NSCLC cell lines *in vitro.* (**a**) Schematic diagram illustrates the experimental design of the pooled RNAi screen to identify genes affecting cell proliferation/survival in H460 and H358. Deep sequencing was used to quantify *TRIM14* shRNA representation in H460 and H358 cell populations at multiple time points for two independent shRNAs. (**b**) Profiling TRIM14 protein expression in a panel of human NSCLC cell lines of different histological subtypes via Western blot analyses. (**c**) Whole-cell extracts from cell lines stably expressing either full-length *TRIM14* or shRNAs against *TRIM14* were subjected to Western blot analysis with anti-TRIM14 antibody and compared with isogenic shGFP or empty vector (EV) controls. β-actin served as a loading control. (**d**) TRIM14 downregulation and overexpression in cells (red) affects cell viability as measured by MTS assays. (**f**) Soft agar was used to assess the ability to form colonies in isogenic cell lines. Results shown represent three biological replicates. (Two-tailed student’s t-test, ****p < 0.0001).

We previously identified a prognostic multigene expression signature for early-stage NSCLC patients and hypothesized that these genes may function in the development and/or progression of NSCLC^10^. To identify proteins affecting the cell proliferation/survival of NSCLC cells, we performed a screen using a library of about 100 short-hairpin RNAs (shRNAs) against the genes in our signature. Briefly, H460 and H358 cells were transfected with individual shRNA oligos from the library, allowed to undergo multiple doubling periods to facilitate stable shRNA integration and expression, and harvested at multiple time points to assess the effect of each shRNA on cell proliferation over time (Fig. 1a). Reduction in the representation of 20 shRNAs corresponding to genes essential for cell viability (e.g. *CDK11B, RPS14*, etc.) showed that the screen was functional (Supplemental Figure 1). Interestingly, downregulation of TRIM14 expression enhanced cell proliferation/survival in both H358 and H460 as represented by hairpin enrichment over time (Fig. 1a).

To validate the functional role of *TRIM14* as a putative tumor suppressor gene (TSG) in lung cancer, we first screened TRIM14 protein expression using Western blot analysis in twelve different NSCLC cell lines and an immortalized but non-tumorigenic human bronchial epithelial cell line (HBE135). TRIM14 protein levels varied substantially among NSCLC cell lines with six out of twelve lines having lower expression levels compared to HBE135 cells (Fig. 1b).

We next established a panel of NSCLC isogenic cell lines that stably expressed the *TRIM14* open reading frame (ORF) or shRNAs against *TRIM14*. H1650, H520, H157 and H358 cell lines, which endogenously express TRIM14, were transduced with two independent shRNAs (shTRIM14.A and shTRIM14.B) or a vector control (shGFP) and downregulation efficiency assessed by quantitative RT-PCR and Western blot analysis (Fig. 1c and Supplemental Figure 2). Both shTRIM14.A and shTRIM14.B mediated a clear decrease in TRIM14 protein levels relative to the vector control. We further overexpressed *TRIM14* ORF constructs in H1395 and H3255 cell lines with nearly undetectable endogenous TRIM14 protein levels (Fig. 1c). Stable integration of shRNA or overexpression constructs did not alter the morphology of the cells, which remained epithelial in appearance.

Using MTS assays, we confirmed a significant increase in cell proliferation of H1650, H520 and H157 upon TRIM14 downregulation, consistent with our initial observation in H460 and H358 cells in the shRNA screen (Fig. 1d; p < 0.0001). Conversely, overexpression of TRIM14 in H1395 and H3255 cells significantly augmented the growth rate of H1395 and H3255 cells (Fig. 1d; p < 0.0001 and p = 0.0025, respectively). Furthermore, the exogenous expression of TRIM14 significantly decreased soft agar colony formation of H1395 cells, while downregulation of TRIM14 increased the number of colonies formed by H1650 and H157 cells (Fig. 1e).

### Loss of TRIM14 contributes to tumorigenicity of NSCLC cell lines *in vivo*

**Figure 2:**
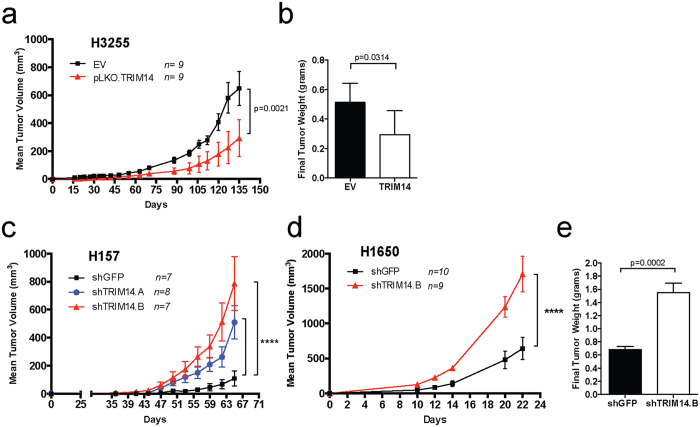
Loss of TRIM14 contributes to NSCLC tumorigenicity in mice. (**a**) NSCLC cells were injected into the left flanks of 6–8 week-old *SCID* mice (n = 7–10 animals per group). Exogenous TRIM14 expression in H3255 significantly suppressed tumor growth in H3255-bearing mice. (**b**) Final tumor weights were measured for each H3255 tumor-bearing mouse at necropsy (EV = empty vector control). (**c–d**) Downregulation of TRIM14 in H157 and H1650 cells (red and blue) significantly increased tumor growth in mice. (**e**) Final tumor weights were measured for each H1650 tumor-bearing mouse at endpoint. (Two-way mixed ANOVA analyses for tumor growth rates and two-tailed student’s t-test for final weight measurements, ****p < 0.0001).

We injected isogenic cell lines into subcutaneous tissue of *scid* mice to assess tumor growth rates. Overexpression of TRIM14 significantly attenuated tumor growth in H3255 xenograft models (Fig. 2a; p = 0.0021). Tumors formed by H3255-TRIM14 cells exhibited significantly reduced tumor weights than tumors formed by the empty vector controls (EV) at necropsy (Fig. 2b; p = 0.0314). Conversely, tumors formed by TRIM14-deficient cells (H1650 and H157) exhibited increased tumor growth rates and greater tumor weights than control tumors (Fig. 2c–e).

**Figure 3:**
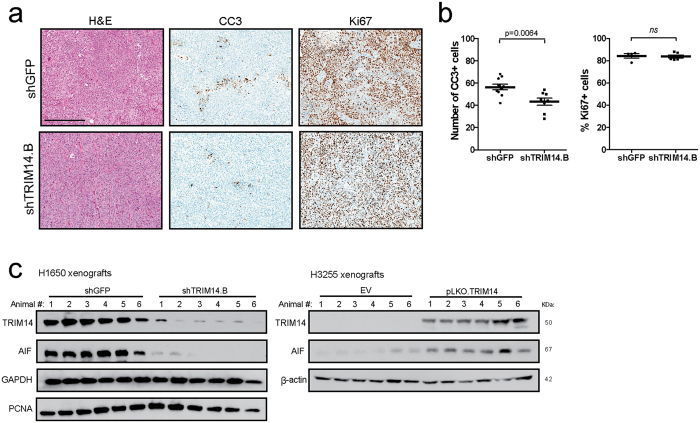
Reduced tumor growth of TRIM14-deficient cells correlates with decreased apoptotic activity *in vivo.* (**a,b**) Representative histologic sections of xenografts of H1650 tumors were immunostained with Ki67 and cleaved-caspase-3 (CC3) antibody and the number of positive cells were quantified for 10 fields at high power (n = 10 controls; n = 8 shTRIM14.B; scale bar  =  300 μm). (**c**) Total extracts from H1650 and H3255 xenograft tumors were subjected to Western blot analysis using the indicated antibodies and compared to isogenic controls. β-actin and GAPDH was used as loading controls. (Abbreviations: EV =  Empty vector; Two-tailed student’s t-test; ****p < 0.0001, *ns:* not significant).

Immunohistochemical (IHC) detection of cell proliferation marker Ki67 in H1650 xenograft tumor sections did not show a significant difference between control and experimental groups (Fig. 3b). However, staining using apoptotic marker cleaved caspase-3 demonstrated a significant albeit modest decrease in the number of cells with active caspase-3 in H1650-shTRIM14 xenograft tumors compared to controls (Fig. 3a,b; p = 0.0064). Moreover, Western blot analyses on tumor lysates confirmed sustained silencing of TRIM14 protein levels in H1650 during tumor progression, which coincided with reduced protein expression of apoptosis-inducing factor (AIF), another caspase-independent apoptotic pathway (Fig. 3c). Given that we observed apoptotic changes rather than expression of Ki67 or proliferating cell nuclear antigen (PCNA), we speculate that *TRIM14* expression mediates apoptotic and cell death signaling. Together these results suggest that TRIM14 behaves as a TSG in NSCLC xenografts.

### Association of *TRIM14* expression with survival of NSCLC patients

We previously identified *TRIM14* as a component of a prognostic multigene expression signature for early-stage NSCLC patients^10^. By analyzing the individual prognostic significance of TRIM14 expression in publicly available gene expression datasets of primary NSCLCs, we found that high TRIM14 mRNA levels in tumors correlate with better survival outcome after surgery, consistent with its antitumor activity (Table 1). The four published microarray studies represented a total of 683 early-stage surgically resected NSCLC patients who had not received adjuvant chemotherapy or radiotherapy. Univariate survival analyses revealed that patients with high *TRIM14* expression survived longer than patients with low expression in the BR.10 patient cohort (hazard ratio [HR] = 0.22, 95% CI = 0.09–0.56, p = 0.002) and the Director’s Challenge Consortium (DCC) cohort (HR = 0.61, 95% CI = 0.59–4.49, p = 0.026). *TRIM14* expression did not correlate with survival in the University of Michigan study, which included only squamous carcinoma patients (HR = 1.17, 95% CI = 0.48–2.83, p = 0.733) (Table 1). Multivariate analysis revealed significant associations between *TRIM14* expression and survival when adjusted for histological subtype, stage, age and sex in DCC and BR.10 cohorts. This data further supports the notion that TRIM14 down-regulation promotes NSCLC progression, which may lead to a poor clinical outcome.

### TRIM14 sensitizes NSCLC cells to anoxic-induced cell death

**Figure 4:**
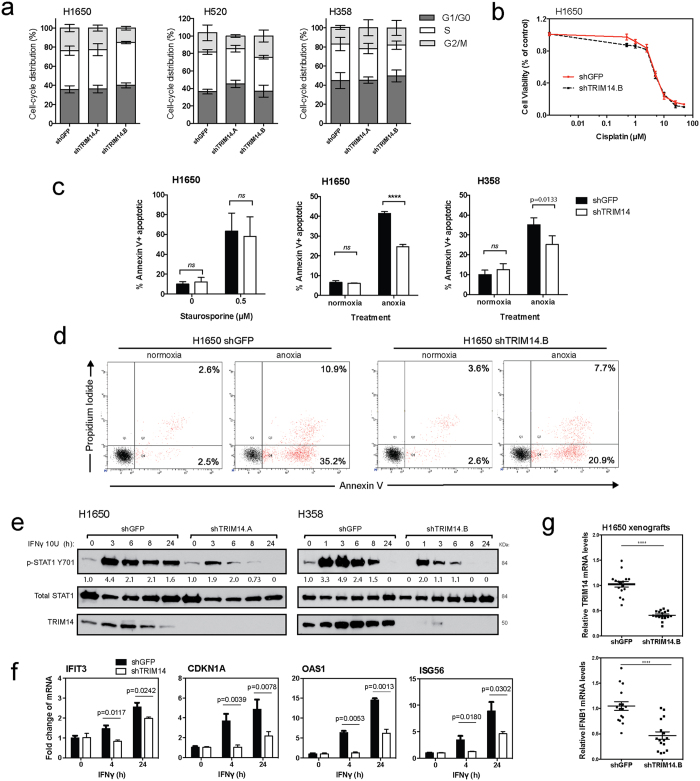
TRIM14 sensitizes NSCLC cells to anoxic-induced cell death and Type II interferon response. (**a**) Cell cycle progression of isogenic cell lines was assessed by flow cytometry after propidium iodide (PI) staining to determine the percent distribution of G1, S or G2/M-phase cell populations. (**b**) MTS assay was used to measure cell viability of H1650 cells treated with serial dilutions of cisplatin for 48 hours. (**c**) H1650 and H358 cells cultured for 48 hours with a protein kinase inhibitor, Staurosporine (0.5 μM), or under anoxic conditions were fixed and stained for Annexin-V and PI. Flow cytometry was subsequently used to quantitate the percentage of Annexin-V positive cells after treatment. (**d**) Representative flow cytometry analysis of H1650 cells cultured with or without anoxic conditions. (**e**) H1650 and H358 cells were treated with or without 10 U IFNγ for indicated times. Phosphorylation of STAT1 at tyrosine 701, total STAT1 and TRIM14 expression were analyzed by immunoblotting. (**f**) Quantitative RT-PCR using RNA extracted at 4 and 24 hours after IFNγ treatment showed increased transcript levels of *ISG56, P21, IFIT1,* and *OAS1* as compared to untreated cells, but this effect was significantly suppressed in TRIM14-deficient cells. (**g**) Quantitative RT-PCR was used to show that mRNA levels for *TRIM14* and *IFNB1* were significantly reduced in H1650 xenograft tumors compared to controls (n = 8 tumors with two technical replicates each). Results shown represent more than three biological replicates. (Two-tailed student’s t-test; ****p < 0.0001).

Given the biological implications of the *in vivo* results, we sought to understand the mechanism of TRIM14’s antitumor activity. We first analyzed the effect of TRIM14 downregulation on cell cycle progression by employing flow cytometry after propidium iodide staining. No significant effect on the distribution of G1, S or G2/M-phase cell populations was observed (Fig. 4a). Next, to determine whether TRIM14 may be inhibiting cell death pathways we assessed the effect of various apoptotic stimuli on TRIM14-deficient cells. While Staurosporine and Cisplatin treatment had no differential apoptotic effects on TRIM14-deficent cells compared to controls, apoptosis was significantly attenuated under anoxic conditions (<0.02% pO_2_ hypoxia) in TRIM14-deficient cells (H1650 and H358), suggesting that these cells were resistant to hypoxia-induced cell death (Fig. 4c,d; p < 0.0001 and 0.0133, respectively; Supplemental Figure 3). Furthermore, downregulation of TRIM14 showed no effect on phosphorylation status of extracellular signal-regulated kinase (ERK) and serine/threonine kinase (AKT), two classic signaling molecules involved in cell proliferation and survival (Supplemental Figure 4). This result suggests that TRIM14 reduces growth of NSCLC cells primarily by affecting cell death pathways, rather than by inhibiting proliferative signaling.

### Loss of TRIM14 attenuates interferon response in NSCLC cells

Because previous studies have implicated the TRIMs as critical regulators in innate immune responses, we hypothesized that TRIM14 may exert antitumor cell effects via interferon signaling pathways in lung cancer cells^8^,^16^. Downregulation of TRIM14 attenuated interferon-γ (IFNγ)-induced activation of signal transducer and activator of transcription 1 (STAT1) phosphorylation at tyrosine 701 in cells over time. Western blot analysis of IFNγ-treated H1650 control cells (10 units/ml [U]) revealed that phospho-STAT1 levels peaked at 1 hour and remained at high levels for up to 24 hours. In contrast, IFNγ treatment of TRIM14-deficient H1650 cells failed to induce sustained phospho-STAT1 levels, which decreased rapidly by 3 hours (Fig. 4e). Similar kinetics of STAT1 activation were observed in H358 cells when treated with IFNγ. These results suggest that TRIM14 is required for STAT1 activation following IFNγ stimulation. TRIM14 downregulation also significantly decreased transcription of well-characterized IFNγ-induced genes and STAT1 downstream targets in H1650 cells upon IFNγ treatment (Fig. 4f). In control cells, quantitative RT-PCR using RNA extracted at 4 and 24 hours after IFNγ treatment showed significantly increased levels of *IFIT3, CDKN1A, OAS1 and ISG56* transcripts as compared to vehicle-treated cells. However, this enhancement was attenuated in the TRIM14-deficient cells indicating that TRIM14 is critical for STAT1-dependent gene expression.

Furthermore, recent studies have reported impaired type I IFN production in TRIM14^−/−^ mice upon herpes simplex (HSV) infection^17^. Quantitative RT-PCR using RNA extracted from H1650 xenografted tumors showed significantly reduced IFN-β1 levels in TRIM14-deficient tumors compared to controls (Fig. 4g; p < 0.0001). Collectively, these results suggest that TRIM14 is a critical regulator in innate immune response and a mediator of IFNγ-induced gene expression via STAT1 signaling.

### Regulation of TRIM14 stability by proteasome degradation

**Figure 5:**
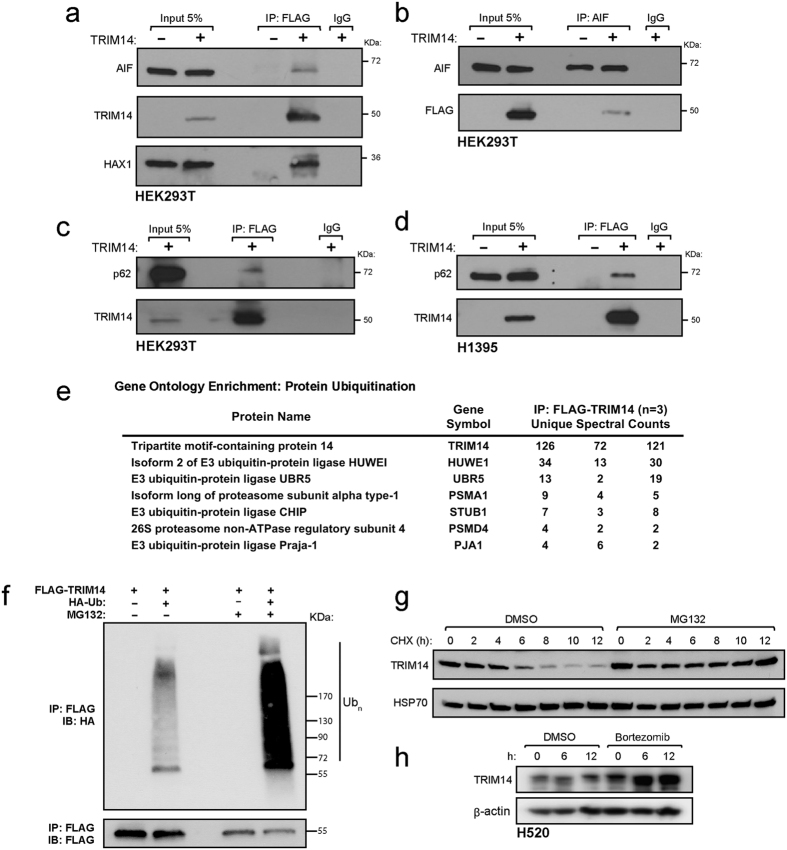
TRIM14 protein stability is regulated by ubiquitin-proteasome system. (**a**) Whole-cell extracts from HEK293T cells transfected with either empty FLAG vector or vector ectopically expressing FLAG-TRIM14 were immunoprecipitated with anti-FLAG antibody, and immune complexes was blotted with indicated antibodies. The ectopically expressed TRIM14 bound with endogenous AIF and HAX1 in HEK293T cells. (**b**) Conversely, endogenous TRIM14 was immunoprecipitated using anti-AIF antibody in HEK293T cells. (**c**) Ectopically expressed FLAG-TRIM14 immunoprecipitated with endogenous p62 in HEK293T and (**d**) H1395 cells. (**e**) Mass spectrometry analysis of FLAG-TRIM14 complexes independently showed that TRIM14 interacted with a number of E3 ubiquitin ligases and proteasome activators in HEK293T cells. (**f**) HEK293T cells were transiently transfected with the indicated vectors and were treated with or without 10 μM MG132 for 4 hours demonstrating that ubiquitylation of FLAG-TRIM14 is elevated in the presence of MG132. (**g**) HEK293T cells were treated with either DMSO or 10 μM MG132 for 4 hours before the addition of 100 μg/ml of cycloheximide (CHX). Immunoblot analysis was performed on HEK293T cell lysates at the indicated times post-cycloheximide treatment to determine TRIM14 protein stability. (**h**) Immunoblot analysis was done on H520 cells treated with either vehicle (DMSO) or Bortezomib (10 nM) at indicated time points. Abbreviations: IgG =  immunoglobulin G, IP = Immunoprecipitation and IB = Immunoblotting.

To identify TRIM14 interacting proteins, we employed immunoprecipitation (IP) coupled with LC-MS/MS. HEK293T cells were transiently transfected with the full length TRIM14 ORF tagged at the C-terminus with 3xFLAG. FLAG-tagged TRIM14 complexes were isolated from total protein lysate by immunoprecipitation 48 hours after transfection and eluted proteins were digested with trypsin and characterized by liquid chromatography-tandem mass spectrometry (LC-MS/MS) using a Protein Prophet p-value cutoff of > 0.95^18^. Specific TRIM-FLAG interactors were identified by excluding proteins that were also pulled down by anti-FLAG beads incubated with total protein lysates from cells transfected with empty FLAG vector (control). Of three biological experiments conducted, only proteins detected in at least two independent runs were further considered. Peptides corresponding to 70 different proteins were detected in 2/3 independent runs and 24 proteins were detected with the highest confidence in 3/3 biological experiments using HEK293T (Table 2). To validate our MS shortlist, we performed co-immunoprecipitation assays to show that TRIM14 bound to AIF and HAX1 in HEK293T cells (Fig. 5a,b). Ectopically expressed FLAG-TRIM14 immunoprecipitated with endogenous p62 in both HEK293T and H1395 cells (Fig. 5c,d). We next identified enriched Gene Ontology (GO) functions in this interactome by performing David bioinformatics (Supplemental Table 5). Enrichment analysis revealed significantly enriched canonical pathways associated with: (1) protein ubiquitination, (2) programmed cell death, and (3) regulation of apoptosis.

Given the abundance of E3 ubiquitin ligases and proteasome activators that consistently immunoprecipitated with TRIM14, we postulate that TRIM14 protein levels may be regulated by the proteasome (Fig. 5e). To examine whether TRIM14 is ubiquitylated *in vivo*, we ectopically expressed both HA-ubiquitin and FLAG-TRIM14 in HEK293T cells in the presence or absence of the proteasome inhibitor MG132. Addition of MG132 enhanced the levels of ubiquitin linked to FLAG-TRIM14 (Fig. 5f). We then examined the effect of proteasome inhibition on the stability of TRIM14. HEK293T cells were treated with either DMSO or MG132 and harvested at indicated time intervals after cycloheximide (CHX) treatment (Fig. 5G). The half-life of TRIM14 was ~6 hours in the absence of proteasome inhibition but was extended to >12 hours in the presence of MG132, indicating that the proteasome is required for rapid turnover of TRIM14 protein levels. Furthermore, treatment with a more potent proteasome inhibitor, Bortezomib, on H520 cells enhanced TRIM14 protein levels after 6 hours on treatment (Fig. 5h).

## Discussion

Here we report the first demonstration of tumor suppressive activity and immune regulation of TRIM14 in lung cancer cells. A pooled shRNA screen targeting genes of a 15-gene signature predictive of prognosis in NSCLC patients^19^ identified TRIM14 as impacting NSCLC cell proliferation/survival. The tumor suppressive function of TRIM14 was subsequently confirmed in several NSCLC cell line models both *in vitro* and *in vivo*.

Supporting our animal studies is the *in silico* finding that low *TRIM14* expression is correlated with worst clinical outcome. Here, we confirmed the individual prognostic value of *TRIM14* expression in the Director’s Challenge and BR.10 expression datasets using Cox-regression analysis^10^,^12^. Both univariate and multivariate survival analysis indicated poorer survival in patients with low *TRIM14* expression, supporting our observation that TRIM14 downregulation promotes NSCLC progression, which may lead to a poor clinical outcome in patients. Little is known about the role of TRIM14 in normal and disease states and it remains unclear how TRIM14 is downregulated in NSCLC and cancers in general. TCGA data reveals rare mutations (0.6–2.9%) and deletions (1%) in either lung adenocarcinoma or squamous carcinoma, suggesting that TRIM14 loss of function in NSCLC may be attributed to epigenetic regulation (http://www.cbioportal.org/). Across human cancer types, *TRIM14* aberrations vary from amplifications in one cancer type and deletions in another, alluding to the possibility of tissue- and cell-type specific roles of TRIM14. Su *et al*.^9^ most recently reported that TRIM14 overexpression promoted tongue squamous cell carcinoma aggressiveness, an opposing phenotype to our observations in lung cancer cells. Interestingly, multivariate analysis also showed that *TRIM14* expression did not correlate with survival in the University of Michigan study, which included only lung squamous carcinoma patients. Given that lung squamous carcinoma and adenocarcinomas are often considered two distinct diseases due to different mutation spectrums, we postulate that TRIM14 may behave differently in the two histological subtypes. Despite these phenotypical discrepancies between the current study and Su *et al*.^9^, both emphasize a pertinent role of aberrant TRIM14 expression in promoting carcinogenesis.

The TRIM family of proteins mediates a variety of biological processes. Alterations in TRIMs are associated with diverse pathological conditions, including developmental disorders, autoimmune disease, viral infections and cancer. A number of TRIMs, such as TRIM13, TRIM8, TRIM19 and TRIM26, function as tumor suppressor proteins by regulating transcriptional activity and apoptosis in a variety of cancer types^20^,^21^,^22^,^23^,^24^. For instance, TRIM19, also known as Promyelocytic leukemia protein (PML), has a tumor suppressor function, and cells derived from PML^−/−^ mice are defective in inducing apoptosis by Fas, TNFα, or interferons^25^. TRIM27 also positively regulates TNF-α-induced apoptosis, and TRIM27^−/−^ mice are resistant to TNF-α cytotoxicity^26^. TRIM16, which along with TRIM14 lacks a RING finger domain, has been shown to reduce neuroblastoma and breast cancer cell growth and migration by activating caspase-2 to induce apoptosis^27^,^28^. In this study, xenografted tumors from TRIM14-deficient cells gave rise to faster tumor growth with markedly reduced apoptotic cells compared to its isogenic controls at necropsy. Because apoptotic pathways other than caspase activation exist, we further confirmed by Western blot analysis that silencing TRIM14 significantly reduced expression of AIF *in vivo*. Moreover, using Annexin-V staining by flow cytometry, we observed that TRIM14 downregulation significantly attenuated anoxia-induced apoptosis in both H1650 and H358 cells. Given that solid tumors often experience hypoxic conditions, we postulate that cells expressing TRIM14 confer sensitivity to apoptotic stimuli as reported for other TRIM protein members, resulting in retarded tumor growth compared to tumors lacking TRIM14^29^,^30^. We also observed that TRIM14 co-immunoprecipitate eluates were enriched for apoptotic-related proteins (Table 2). In fact, AIF was among the proteins detected by mass spectrometry experiments and validated to bind with TRIM14 by independent co-immunoprecipitation assays (Fig. 5a,b). Indeed, previous studies showed that TRIM14 is localized to the outer mitochondria membrane and AIF is released from the mitochondria to induce apoptosis signaling cascades^7^,^31^. How TRIM14 engages AIF in NSCLC remains unclear, but our results raise the possibility that caspase-independent cell death pathways may partly mediate TRIM14 tumor suppressive function. Collectively, our findings support the notion that TRIM14 may be acting via apoptotic/cell death pathways to exert its tumor suppressive phenotype. Further studies on the exact mechanism by which TRIM14 mediates these pathways will be of great interest.

A systematic analysis of all 75 known human TRIMs revealed that nearly half of these proteins could enhance the innate immune response^32^. Emerging evidence suggests that the outgrowth of cancer cells into clinically detectable tumors requires immunoediting to reduce cellular immunogenicity and to escape the immune recognition that otherwise would lead to cancer cell elimination or growth inhibition^33^. Among the many immunoregulatory cytokines, the type II IFNs are potent regulators of most effector cells involved in anti-tumor immune responses^33^. Here we demonstrated for the first time that TRIM14 positively regulates type II IFN signaling in lung cancer cells. Silencing TRIM14 attenuated STAT1 pathway activation induced by IFNγ in a time-dependent manner. Moreover, we showed that TRIM14 downregulation significantly deceased the mRNA levels of well-known STAT1 downstream targets, such as *CDKN1, ISG56* and *OAS1* upon IFNγ induction. Earlier studies on TRIM19 showed that IFNγ-mediated STAT1 DNA-binding activity and apoptosis were reduced in PML^−/−^ mouse embryonic fibroblasts (MEFs) compared with wild-type MEFs^34^. Until recently, Chen *et al*.^17^ demonstrated that TRIM14^−/−^ mice were highly susceptible to HSV-1 infection due to impaired type I IFN production. Mechanistically, the authors revealed that TRIM14 stabilizes cyclic GMP-AMP synthase (cGAS), a DNA sensor that triggers IFN signaling, by inhibiting cGAS degradation via selective autophagy receptor p62^17^. Here, we demonstrate for the first time that p62 (*SQSTM1*) immunoprecipitates with TRIM14 in both HEK293T and H1395 lung cancer cells (Fig. 5c,d). Such co-immunoprecipitation may be due to multi-component TRIM14-cGAS-p62 complex pull-down or an unexplored mechanism by which TRIM14 stabilizes cGAS by blocking p62. Moreover, we also show that IFN-β1 mRNA levels are significantly reduced in TRIM14-deficient xenograft tumors compared to controls (Fig. 4g). Given that prior studies have demonstrated that STAT1 controls anti-tumorigenic effects in part by upregulation of caspases, p21 and the IFN-regulated factor 1 (IRF1) pathway, we postulate that lung cancer cells may suppress TRIM14 to promote tumorigenicity through immune escape^35^,^36^. It would be interesting to examine whether cells with TRIM14 expression in cancer cells are more susceptible to IFN anti-proliferative effects in immunocompetent animal models.

In conclusion, our findings provide the first *in vitro* and *in vivo* evidence that TRIM14 has a tumor suppressive role in lung cancer. Consistent with our initial genetic screen, TRIM14 expression in clinical samples correlated with poorer survival outcome. Overexpression of TRIM14 reduced cell viability, tumorigenicity, apoptosis as well as Type II interferon response. Our results provide new insights into the pathogenesis of NSCLC and highlight TRIM14 as a potential tumor suppressor and regulator of innate immune response in NSCLC cells.

## Additional Information

**How to cite this article**: Hai, J. *et al*. TRIM14 is a Putative Tumor Suppressor and Regulator of Innate Immune Response in Non-Small Cell Lung Cancer. *Sci. Rep.* **7**, 39692; doi: 10.1038/srep39692 (2017).

**Publisher's note:** Springer Nature remains neutral with regard to jurisdictional claims in published maps and institutional affiliations.

## Supplementary Material

Supplementary Information

## Figures and Tables

**Table 1:** Clinical correlation of *TRIM14* expression in NSCLC patients.

Cohort	Univariate Survival Analysis			*TRIM14* (probe set: 203147_s_at)		
	Tumor Type	Platform	No. patients	Hazard Ratio	95% CI	P-value
JBR.10	NSCLC	U133A	62	**0.22**	0.09–0.56	**0.002**
DCC	ADC	U133A	311	**0.61**	0.40–0.94	**0.026**
UHN	NSCLC	U133A plus2.0	181	1.48	0.98–2.22	0.063
Michigan	SQC	U133A	129	1.17	0.48–2.83	0.733
	**Multivariate Survival Analysis**			***TRIM14***** (probe set: 203147_s_at)**		
JBR.10	NSCLC	U133A	62	**0.23**	0.08–0.66	**0.006**
DCC	ADC	U133A	311	**0.61**	0.40–0.95	**0.030**
UHN	NSCLC	U133A plus2.0	181	1.41	0.93–2.14	0.111
Michigan	SQC	U133A	129	1.19	0.49–2.89	0.706

Abbreviations: DCC, Director’s Challenge Consortium adenocarcinoma; NSCLC, non-small cell lung cancer; UHN, University Health Network; U133A, Affymetrix U133A chip; ADC, adenocarcinoma; SQC, squamous cell carcinoma.

*Hazard ratio compares the overall survival of the high-risk (poor prognosis) patient group to that of the low-risk (good prognosis) group.

**Table 2:** Top 70 proteins that co-immunoprecipitated with TRIM14 in HEK293T cells.

	Identified Proteins (70)	Gene Symbol	Empty vector Exp.			TRIM14 Exp.		
			1	2	3	1	2	3
1	Tripartite motif-containing protein 14	TRIM14	0	0	0	126	72	121
2	DNA-dependent protein kinase catalytic subunit	PRKDC	0	0	0	8	3	75
3	*Isoform 2 of E3 ubiquitin-protein ligase HUWEI*	HUWE1	0	0	0	34	13	30
4	Isoform 5 of Protein transport protein	SEC16A	0	0	0	9	23	21
5	CAD protein	CAD	0	0	0	13	4	29
6	*E3 ubiquitin-protein ligase UBR5*	UBR5	0	0	0	13	2	19
7	Isoform 2 of Melanoma-associated antigen D1	MAGED1	0	0	0	13	15	13
8	DnaJ homolog subfamily A member 2	DNAJA2	0	0	0	5	4	12
9	Melanoma-associated antigen D2	MAGED2	0	0	0	3	13	6
10	Translational activator GCN1	GCN1L1	0	0	0	11	2	18
11	Sequestosome-1 p62	SQSTM1	0	0	0	11	6	9
12	Bifunctional glutamate/proline--tRNA ligase	EPRS	0	0	0	4	2	13
13	Serine/threonine-protein phosphatase 6 regulatory subunit 1	PPP6R1	0	0	0	8	3	4
14	*Isoform long of proteasome subunit alpha type-1*	PSMA1	0	0	0	9	4	5
15	Isoform 2 of HCLS1-associated protein X-1	HAX1	0	0	0	4	5	6
16	Isoform 2 of Protein CIP2A	KIAA1524	0	0	0	4	2	4
17	*E3 ubiquitin-protein ligase CHIP*	STUB1	0	0	0	7	3	8
18	RuvB-like 2	RUVBL2	0	0	0	6	5	8
19	*26S proteasome non-ATPase regulatory subunit 4*	PSMD4	0	0	0	4	2	2
20	*E3 ubiquitin-protein ligase Praja-1*	PJA1	0	0	0	4	6	2
21	Structural maintenance of chromosomes protein	SMC4	0	0	0	2	2	4
22	Isoform 2 of A-kinase anchor protein 12	AKAP12	0	0	0	3	3	2
23	Programmed cell death protein 5	PDCD5	0	0	0	2	4	2
24	Nucleosome assembly protein 1-like 4 (Fragment)	NAP1L4	0	0	0	4	3	2
25	*Proteasomal ubiquitin receptor ADRM1*	ADRM1	0	0	0	4	0	2
26	A-kinase anchor protein 8-like	AKAP8L	0	0	0	19	8	0
27	Apoptosis-inducing factor 1, mitochondrial	AIFM1	0	0	0	0	2	20
28	DnaJ homolog subfamily C member 7	DNAJC7	0	0	0	12	6	0
29	Tubulin beta-8 chain	TUBB8	0	0	0	6	0	3
30	Immunoglobulin-binding protein 1	IGBP1	0	0	0	5	0	3
31	Cancer/testis antigen family 45 member A5	CT45A5	0	0	0	7	7	0
32	Tubulin beta-2A chain	TUBB2A	0	0	0	4	0	6
33	Golgi-specific brefeldin A-resistance guanine nucleotide exchange factor 1	GBF1	0	0	0	0	2	5
34	DnaJ homolog subfamily B member 1	DNAJB1	0	0	0	4	0	4
35	HEAT repeat-containing protein 6	HEATR6	0	0	0	3	0	3
36	Structural maintenance of chromosomes protein 2	SMC2	0	0	0	2	0	4
37	Serine/threonine-protein phosphatase 6 regulatory subunit 3	PPP6R3	0	0	0	3	0	4
38	*Proteasome subunit alpha type-7*	PSMA7	0	0	0	4	0	2
39	Prefoldin subunit 2	PFDN2	0	0	0	0	5	2
40	La Ribonucleoprotein Domain Family Member 4B	LARP4B	0	0	0	2	7	0
41	*Proteasome 26S Subunit, ATPase 6*	PSMC6	0	0	0	5	0	3
42	Tubulin beta-4A chain	TUBB4A	0	0	0	4	0	3
43	Isoform 1 of Fanconi anemia group I protein	FANCI	0	0	0	2	0	3
44	Pyridoxal-dependent decarboxylase domain-containing protein 1	PDXDC1	0	0	0	0	2	6
45	Pre-mRNA-processing-splicing factor 8	PRPF8	0	0	0	2	0	4
46	Isoform 2 of SAM domain and HD domain-containing protein 1	SAMHD1	0	0	0	2	0	6
47	Pumilio homolog 1	PUM1	0	0	0	0	3	3
48	S-phase kinase-associated protein 1	SKP1	0	0	0	2	0	2
49	Isoform 2 of Cdc42 effector protein 1	CDC42EP1	0	0	0	0	3	2
50	Tubulin beta-6 chain	TUBB6	0	0	0	3	0	3
51	Interleukin-1 receptor-associated kinase 1	IRAK1	0	0	0	0	3	7
52	Methionyl-tRNA synthetase	MARS	0	0	0	2	0	5
53	Rho guanine nucleotide exchange factor 2	ARHGEF2	0	0	0	2	4	0
54	BAG family molecular chaperone regulator 2	BAG2	0	0	0	4	0	3
55	Isoform 2 of Caseinolytic peptidase B protein homolog	CLPB	0	0	0	4	0	2
56	Acidic leucine-rich nuclear phosphoprotein 32 family member A	ANP32A	0	0	0	2	2	0
57	Reticulocalbin-1	RCN1	0	0	0	2	2	0
58	Cysteine and glycine-rich protein 2	CSRP2	0	0	0	2	4	0
59	Heat shock 70 kDa protein 4L	HSPA4L	0	0	0	0	2	2
60	Protein SEC13 homolog	SEC13	0	0	0	2	0	2
61	RNA polymerase II-associated protein 1	RPAP1	0	0	0	2	0	4
62	*26S proteasome non-ATPase regulatory subunit 6 f*	PSMD6	0	0	0	2	0	2
63	Mitotic-spindle organizing protein 2A	MZT2A	0	0	0	0	2	2
64	*Proteasome subunit alpha type-2*	PSMA2	0	0	0	2	0	2
65	Isoform 2 of RING finger protein 126	RNF126	0	0	0	2	0	2
66	Tubulin alpha-1C chain	TUBA1C	0	0	0	2	0	2
67	DNA damage-binding protein 1	DDB1	0	0	0	2	0	2
68	DNA polymerase delta subunit 3	POLD3	0	0	0	0	2	2
69	*Proteasome activator complex subunit 3*	PSME3	0	0	0	3	0	2
70	*Proteasome subunit alpha type-4*	PSMA4	0	0	0	3	0	2
71	Mitotic spindle-associated MMXD complex subunit MIP18	FAM96B	0	0	0	2	0	2

Spectral counts for each of the three biological replicates are shown. Proteins in *italic* are involved in proteasome degradation.
