# Supplementary material for: TRIM14 is a Putative Tumor Suppressor and Regulator of Innate Immune Response in Non-Small Cell Lung Cancer
Source: Sci Rep. 2017 Jan 6;7:39692. doi: 10.1038/srep39692 (PMC5216374; doi:10.1038/srep39692)
Supplement: Supplementary Information [file srep39692-s1.pdf]

TRIM14 is a Putative Tumor Suppressor and Regulator of Innate Immune Response in Non-Small Cell Lung Cancer

Josephine Hai[1,2,3,4], Chang-Qi Zhu[1], Tao Wang[1], Shawna L Organ[1], Frances A Shepherd[1] and Ming-Sound Tsao*[1,2]

[1]Princess Margaret Cancer Centre, University Health Network, Toronto, ON, Canada

[2]Department of Medical Biophysics, University of Toronto, Toronto, ON, Canada

[3]Department of Medical Oncology, Dana-Farber Cancer Institute, Boston, MA, USA

[4]Department of Medicine, Harvard Medical School, Boston, MA, USA

Supplemental Methods

Supplemental Tables 1-6

Supplemental Figures 1-8

Supplemental Methods

Quantitative real-time PCR analysis

Total cellular RNA was extracted from xenograft tumours and cell lines using TRIzol reagent (Invitrogen, Burlington, ON) and RNeasy Mini kit (Qiagen, Mississauga, ON), respectively. One microgram of total RNA was reverse transcribed using Superscript II RNase H reverse transcription kit (Invitrogen, Burlington, ON). Real-time qPCR was performed using 10ng of the first-strand cDNA synthesis as a template using Stratagene MX3000P (La Jolla, CA) and the SYBR Green technique. mRNA copy number for was adjusted by the geometric mean of the 3 house-keeping genes (*ACTB, BAT1* and *RPS13*) as previously described[1]. The following are the human specific primers used in this study:

ACTB	Forward	TCCTAAAAGCCACCCCACTTCT
ACTB	Reverse	GGGAGAGGACTGGGCCATT
BAT1	Forward	TGCCTCGGCCAAATAGGTT
BAT1	Reverse	CGGTATCAGCAGTTTAAAGATTTTCA
IFIT3	Reverse	CAGTTGTGTCCACCCTTCCT
IFIT3	Forward	GAACATGCTGACCAAGCAGA
IFNB1	Forward	AGCAGTCTGCACCTGAAAAGATATT
IFNB1	Reverse	TGTACTCCTTGGCCTTCAGGTAA
ISG56	Forward	TTCGGAGAAAGGCATTAGA
ISG56	Reverse	TCCAGGGCTTCATTCATAT
OAS1	Forward	ACAGGCAGAAGAGGACTGGA
OAS1	Reverse	TAGAAGGCCAGGAGTCAGGA
P21	Forward	TGGGGATGTCCGTCAGAAC
P21	Reverse	GGCGTTTGGAGTGGTAGAAATC
RPS13	Forward	GTTGCTGTTCGAAAGCATCTTG
RPS13	Reverse	AATATCGAGCCAAACGGTGAA
TRIM14	Forward	TCACAGCTCCCTCCAGAAGC
TRIM14	Reverse	TTGGGAGAGGGTCAGGAGTAG
TRIM14	Forward2	ACGGCCACCGAGCAGGAGAT
TRIM14	Reverse2	GGCGTGCGCGCGTATTTCAG

Scoring of shRNA pooled screen

To incorporate measurements from multiple time points in an shRNA screen, we used the shARP scoring as previously described by Marcotte *et al.*[2]. The shARP scores were determined for each of the hairpins in the library and used to subsequently calculate the GARP score by averaging the 2 lowest shARP scores. Rather than bootstrapping the small sample size of hairpins to assign significance, the GARP scores were used to rank genes to determine overall hairpin enrichment and depletion.

Immunoprecipitation for liquid chromatography-tandem mass spectrometry (LC MS/MS) analysis

MS Data Analysis Tandem mass spectra were extracted by BioWorks version 3.3. All MS/MS samples were analyzed using SEQUEST (ThermoFinnigan, San Jose, CA; version 27, revision 12) and X! Tandem (The Global Proteome Machine Organization; version 2006.04.01.2). Both search engines were set up to search the Human Uniprot Database dated Sept 5 2012 and containing 86,582 protein sequences. Scaffold (Proteome Software Inc., Portland, OR; version Scaffold-01.06.05) was used to validate MS/MS-based peptide and protein identifications. Peptide identifications were accepted if they could be established at greater than 95.0% probability as specified by the Peptide Prophet algorithm [3]. Proteins that contained similar peptides and could not be differentiated based on MS/MS analysis alone were grouped to satisfy the principles of parsimony.

Supplemental Tables:

Supplemental Table 1. Target sequences and clone IDs per hairpin used in the RNAi screen

	Gene Name	Gene Source ID	Target Seq	TRC Clone ID
**1**	ATP1B1	481	GCCGTACAGTTCACCAATCTT	TRCN0000043333
**2**	ATP1B2	481	GCTCACCATCAGTGAATTTAA	TRCN0000043334
**3**	ATP1B3	481	CCCAAGAGCTATGAGGCATAT	TRCN0000043335
**4**	ATP1B4	481	GTGATGAAGTATAACCCAAAT	TRCN0000043336
**5**	ATP1B5	481	CGGTGGCAGTTGGTTTAAGAT	TRCN0000043337
**6**	L1CAM-1	3897	CCACTTGTTTAAGGAGAGGAT	TRCN0000063913
**7**	L1CAM-2	3897	GCTAACCTGAAGGTTAAAGAT	TRCN0000063914
**8**	L1CAM-3	3897	CCTTTAGGGTTACTGCCATAA	TRCN0000063915
**9**	L1CAM-4	3897	ACGGGCAACAACAGCAACTTT	TRCN0000063916
**10**	L1CAM-5	3897	GCCAATGCCTACATCTACGTT	TRCN0000063917
**11**	HEXIM1	10614	CCCTATTCTAAATCGCTTAAA	TRCN0000245065
**12**	HEXIM2	10614	GTTTGGAGACTAGACTGAAAC	TRCN0000245064
**13**	HEXIM3	10614	GCGGCATTGGAAACCGTACTA	TRCN0000245062
**14**	HEXIM4	10614	ACACCAGCGATGACGACTTCA	TRCN0000245063
**15**	HEXIM5	10614	AGCCTCAAACTAGCAACTGTA	TRCN0000245061
**16**	MDM1	4193	CTTTGGTAGTGGAATAGTGAA	TRCN0000003376
**17**	MDM2	4193	GATTCCAGAGAGTCATGTGTT	TRCN0000003377
**18**	MDM3	4193	CGATTATATGATGAGAAGCAA	TRCN0000003378
**19**	MDM4	4193	CTGTGTGTAATAAGGGAGATA	TRCN0000003379
**20**	MDM5	4193	CTCAGCCATCAACTTCTAGTA	TRCN0000003380
**21**	IKB1	8518	GCGTCAAATATCACGTCATTT	TRCN0000037869
**22**	IKB2	8518	CCCAAAGAATATCTTCCATTT	TRCN0000037870
**23**	IKB3	8518	CGGTTCTAGGTCCCAATTCTA	TRCN0000037871
**24**	IKB4	8518	GCCAGATATTTAAGTACCTTT	TRCN0000037872
**25**	IKB5	8518	GCTGTGCTCTTGCTGTTAGAA	TRCN0000037873
**26**	TRIM1	9830	GCCCGTCAAGAGCTTCTTTAA	TRCN0000061828
**27**	TRIM2	9830	GCGATCGCTATTGCTGAAATA	TRCN0000061829

**28**	TRIM3	9830	GCTAATGCAGAGTCAAGTAAA	TRCN0000061830
**29**	TRIM4	9830	GCCATTGGACATTCGCCTTAA	TRCN0000061831
**30**	TRIM5	9830	CTCAGATTACTACTTGACGAA	TRCN0000061832
**31**	MLANA1	2315	GCCAGAGGTAATGTTAGTAAA	TRCN0000128356
**32**	MLANA2	2315	GCTTATGAGAAACTCTCTGCA	TRCN0000129054
**33**	MLANA3	2315	CGCCACTATGCCTGACTAATT	TRCN0000128101
**34**	MLANA4	2315	CAAAGTGTCTCTTCAAGAGAA	TRCN0000129699
**35**	MLANA5	2315	CCAGAAATTGGTAGAAGGATT	TRCN0000128521
**36**	MTYL1	23040	CCGATATGATTAAACTCAGAA	TRCN0000020864
**37**	MTYL2	23040	CCTCGTTTGAATACAACAGTT	TRCN0000020865
**38**	MTYL3	23040	CGAAAGCCATTTGCCGTGAAA	TRCN0000020866
**39**	MTYL4	23040	GCAGCAAGACAGTAGAAATAT	TRCN0000020867
**40**	MTYL5	23040	CCATCGCTTTGGAAACGGAAA	TRCN0000020868
**41**	FOS1	2355	GCAGTGAGTATTGGAAGACTT	TRCN0000016138
**42**	FOS2	2355	CAGCAGAAATTCCGGGTAGAT	TRCN0000016139
**43**	FOS3	2355	GCGCTCTGTCATCAAGCCCAT	TRCN0000016140
**44**	FOS4	2355	CACCTCCATGTCCAACCCATA	TRCN0000016141
**45**	FOS5	2355	CACGGCCCAGTGTGCAAGATT	TRCN0000016142
**46**	MB1	4151	CCAGAGACTCTGGAGAAGTTT	TRCN0000059283
**47**	MB2	4151	ACCTGGAGTTCATCTCGGAAT	TRCN0000059284
**48**	MB3	4151	GAAGGCGTCTGAGGACTTAAA	TRCN0000059285
**49**	MB4	4151	GCATCATGAGGCAGAGATTAA	TRCN0000059286
**50**	EDN1	1908	CCTTGATGTTTGTGACAAGAA	TRCN0000117872
**51**	EDN2	1908	GTTGAAGTCAAGGACCAACAA	TRCN0000117873
**52**	EDN3	1908	ACAAGGAGTGTGTCTACTATT	TRCN0000117874
**53**	EDN4	1908	GTAATTCAAGGACGGCAGAAA	TRCN0000117875
**54**	EDN5	1908	CCTATGGACTGTCCAACTACA	TRCN0000117876
**55**	UMP1	7372	CAGATGCTTTAGGACCTAGTA	TRCN0000034816
**56**	UMP2	7372	CGTCTTGTAGAAGGAACTATT	TRCN0000034814
**57**	UMP3	7372	GAAGCGTATTTGAGTAGACTT	TRCN0000034815
**58**	UMP4	7372	GCCTTATACAGCTTTGCCATT	TRCN0000034817
**59**	UMP5	7372	CAGTACAATAGCCCACAAGAA	TRCN0000034818
**60**	FAM1	54478	CCCACCCATTACGGCGATCAA	TRCN0000062588
**61**	FAM2	54478	CTGTCCCAAGAGCTAGATGAA	TRCN0000062589
**62**	FAM3	54478	TCGCTCAGCTAAGAGTGCTTT	TRCN0000062590
**63**	FAM4	54478	CCTGCCAAAGTGGCACCAAGT	TRCN0000062591
**64**	FAM5	54478	GACATCGTCTCTCTCATTCAT	TRCN0000062592
**65**	ZNF1	7776	CGACACACTTACCGCATCAAT	TRCN0000005504
**66**	ZNF2	7776	CGTCCCTATATGTGTCCCTAT	TRCN0000005505
**67**	ZNF3	7776	CCCTGTGTGTAACAAGAAATT	TRCN0000005506
**68**	ZNF4	7776	GCTAACTTGGTTGGACCAAAT	TRCN0000005507
**69**	ZNF5	7776	CCAATCCTCATAACTGACTTA	TRCN0000005508
**70**	STM1	11075	GAACTCCAGGTTGAACTGTCT	TRCN0000140472
**71**	STM2	11075	GTCACTGATCTGCTCTTGCTT	TRCN0000140630
**72**	STM3	11075	GTGAGGCTAATCTAGCTGCTA	TRCN0000140826
**73**	STM4	11075	CAAATCAACAAACGTGCCTCT	TRCN0000141246
**74**	STM5	11075	GCTGAAACAATTGGCAGAGAA	TRCN0000141795
**75**	GFP1	clonetechGfp_228s1c1	CGACCACATGAAGCAGCACGA	TRCN0000072179
**76**	GFP2	clonetechGfp_437s1c1	ACAACAGCCACAACGTCTATA	TRCN0000072181
**77**	GFP3	clonetechGfp_587s1c1	TGCCCGACAACCACTACCTGA	TRCN0000072186
**78**	RFP1	rfp_402s1c1	CGTAATGCAGAAGAAGACCAT	TRCN0000072210

**79**	RFP2	rfp_576s1c1	CTACAAGACCGACATCAAGCT	TRCN0000072219
**80**	RAN	5901	ACGTCATTTGACTGGTGAATT	TRCN0000047929
**81**	EFTUD2	9343	GCTTTGCTGAAACGCCTAATA	TRCN0000074655
**82**	PRPF8	10594	CCCAACTTGTACCGCTACATA	TRCN0000075108
**83**	WBP11	51729	GCTTCCATTGTCAGGGTATTT	TRCN0000074473
**84**	PSMB2	5690	CGCACTCTTGATAAATGGTTA	TRCN0000003907
**85**	RPL9	6133	GACTTCAATCACATCAATGTA	TRCN0000117564
**86**	PRPF38A	84950	CCTTGACTGTATTCAAACTTA	TRCN0000074908
**87**	PSMD8	5707	GCATCATCATTCTGAAGGATA	TRCN0000058107
**88**	RPL32	6161	GCTGATGTGCAACAAATCTTA	TRCN0000007955
**89**	HNRNPC	3183	GCGCTTGTCTAAGATCAAATT	TRCN0000006644
**90**	CDK11B	984	CGGCCTCAAGCATGAGTATTT	TRCN0000006209
**91**	AQR	9716	CGTGCAGATGTTACCATAAAT	TRCN0000074872
**92**	SF3A1	10291	CGTACTGACATCTTCGGTGTA	TRCN0000006600
**93**	PSMA1	5682	CTGCTGATGCTAGACTGTTAT	TRCN0000003872
**94**	SNRPD2	6633	CATCAACTGCCGCAACAATAA	TRCN0000074400
**95**	PSMD1	5707	GCATCATCATTCTGAAGGATA	TRCN0000058107
**96**	SNRPD1	6632	CCTAAGGTGAAATCTAAGAAA	TRCN0000005381
**97**	RPS29	6161	GCTGATGTGCAACAAATCTTA	TRCN0000007955
**98**	RPS14	6208	GCTGAAGGAGAGAATGTATTT	TRCN0000008641

Table 2. Ranking hairpin enrichment based on GARP scores

H460			H358		
**Rank**	**Gene**	**GARP Scores**	**Rank**	**Genes**	**GARP Scores**
1	MLANA	0.068	1	STMN2	0.040011799
2	MYT1L	0.062	2	FOSL2	0.009834825
3	TRIM14	0.037	3	TRIM14	-0.007691474
4	STMN2	0.029	4	ZNF236	-0.009782804
5	ZNF236	-0.080	5	MLANA	-0.013716136
6	IKBKAP	-0.108	6	FAM64A	-0.013994348
7	MDM2	-0.116	7	MYT1L	-0.015844925
8	FOSL2	-0.119	8	ATP1B1	-0.050984448
9	L1CAM	-0.124	9	IKBKAP	-0.074913897
10	EDN3	-0.157	10	MDM2	-0.076451096
11	HEXIM1	-0.159	11	UMPS	-0.115507228
12	ATP1B1	-0.211	12	EDN3	-0.11876518
13	FAM64A	-0.234	13	L1CAM	-0.141716081
14	UMPS	-0.281	14	HEXIM1	-0.205186881
15	MB	-0.288	15	MB	-0.212918085

Supplemental Table 3. Prognostic value of *TRIM14* expression compared with other clinical and pathologic variables.

	Multivariate Survival Analysis		
Variables	HR	95% CI	P-value
JBR.10 [4]			
TRIM14 expression	0.23	0.08-0.66	0.006
Stage III vs. II vs. I	2.07	0.91-4.72	0.025
Histology (Others vs. ADC)	1.01	0.56-1.84	0.962
Sex (female vs. male)	0.86	0.28-2.58	0.785
Age ≥65 years	2.45	1.00-5.96	0.049
DCC [5]			
TRIM14 expression	0.61	0.40-0.95	0.030
Stage III vs. II vs. I	2.19	1.74-2.76	<0.0001
Histology (others vs. ADC)	-	-	-
Sex (female vs. male)	0.78	0.51-1.18	0.241
Age ≥65 years	1.03	0.70-1.54	0.870
Michigan [6]			
TRIM14 expression	1.19	0.49-2.89	0.706
Stage III vs. II vs. I	1.13	0.75-1.70	0.564
Histology (others vs. ADC)	-	-	-
Sex (female vs. male)	1.16	0.60-2.22	0.662
Age ≥65 years	0.91	0.48-1.71	0.766
UHN [7]			
TRIM14 expression	1.41	0.93-2.14	0.111
Stage III vs. II vs. I	1.64	0.94-2.85	0.081
Histology (others vs. ADC)	0.66	0.35-1.26	0.209
Sex (female vs. male)	0.48	0.27-0.84	0.010
Age ≥65 years	1.66	0.89-3.10	0.112

Abbreviations: HR, hazard radio; CI, confidence interval

Supplemental Table 4. Mutational status of human cell line models.

Model	Type	MDM2	TP53	EGFR	KRAS	BRAF
**H3255**	ADC	n/a	WT	L858R	G13C	WT
**H1395**	ADC	Amp	WT	WT	WT	G469A
**H157**	SQC	n/a	c.892G>T	WT	G12R	WT
**H1650**	ADC	n/a	c.673-2A>G	E746_A750del	WT	WT
**H358**	ADC	n/a	Deletion	WT	G12V	WT

Supplemental Table 5. Functional annotation of top TRIM14 binding candidates

GO Term	Count	%	Enrichment	P-value
GO:0031398~positive regulation of protein ubiquitination	10	14	28	5.3E-11
GO:0006511~ubiquitin-dependent protein catabolic process	13	19	13	2.3E-10
GO:0031396~regulation of protein ubiquitination	10	14	24	2.6E-10
GO:0051437~positive regulation of ubiquitin-protein ligase activity during mitotic cell cycle	9	13	31	3.1E-10
GO:0051443~positive regulation of ubiquitin-protein ligase activity	9	13	31	4.0E-10
GO:0051439~regulation of ubiquitin-protein ligase activity during mitotic cell cycle	9	13	30	4.5E-10
GO:0051351~positive regulation of ligase activity	9	13	29	5.6E-10
GO:0051438~regulation of ubiquitin-protein ligase activity	9	13	27	9.6E-10
GO:0051340~regulation of ligase activity	9	13	26	1.3E-09
GO:0031145~anaphase-promoting complex-dependent proteasomal ubiquitin-dependent protein catabolic process	8	11	29	8.2E-09
GO:0051436~negative regulation of ubiquitin-protein ligase activity during mitotic cell cycle	8	11	29	8.2E-09
GO:0010498~proteasomal protein catabolic process	9	13	21	8.3E-09
GO:0043161~proteasomal ubiquitin-dependent protein catabolic process	9	13	21	8.3E-09
GO:0051444~negative regulation of ubiquitin-protein ligase activity	8	11	28	1.0E-08
GO:0051352~negative regulation of ligase activity	8	11	28	1.0E-08
GO:0031397~negative regulation of protein ubiquitination	8	11	26	2.1E-08
GO:0000278~mitotic cell cycle	13	19	8	2.8E-08
GO:0019941~modification-dependent protein catabolic process	15	21	6	5.9E-08
GO:0043632~modification-dependent macromolecule catabolic process	15	21	6	5.9E-08
GO:0031401~positive regulation of protein modification process	10	14	13	6.6E-08
GO:0051603~proteolysis involved in cellular protein catabolic process	15	21	6	1.0E-07
GO:0044257~cellular protein catabolic process	15	21	6	1.1E-07
GO:0044265~cellular macromolecule catabolic process	16	23	5	1.6E-07
GO:0030163~protein catabolic process	15	21	6	1.6E-07
GO:0009057~macromolecule catabolic process	16	23	5	4.1E-07
GO:0032270~positive regulation of cellular protein metabolic process	10	14	10	4.3E-07
GO:0031400~negative regulation of protein modification process	8	11	16	5.5E-07
GO:0051247~positive regulation of protein metabolic process	10	14	10	6.1E-07
GO:0022402~cell cycle process	13	19	5	2.6E-06
GO:0031399~regulation of protein modification process	10	14	8	3.0E-06
GO:0032268~regulation of cellular protein metabolic process	12	17	6	3.1E-06
GO:0006457~protein folding	8	11	11	7.9E-06
GO:0032269~negative regulation of cellular protein metabolic process	8	11	11	8.8E-06
GO:0051248~negative regulation of protein metabolic process	8	11	10	1.1E-05
GO:0007049~cell cycle	14	20	4	1.3E-05
GO:0051258~protein polymerization	5	7	24	4.9E-05

GO:0044092~negative regulation of molecular function	9	13	6	6.4E-05
GO:0006508~proteolysis	15	21	3	7.3E-05
GO:0043086~negative regulation of catalytic activity	8	11	7	1.4E-04
GO:0010604~positive regulation of macromolecule metabolic process	13	19	4	1.6E-04
GO:0043085~positive regulation of catalytic activity	10	14	5	2.6E-04
GO:0043623~cellular protein complex assembly	6	9	9	5.4E-04
GO:0044093~positive regulation of molecular function	10	14	4	6.2E-04
GO:0007018~microtubule-based movement	5	7	11	1.2E-03
GO:0034622~cellular macromolecular complex assembly	7	10	5	1.9E-03
GO:0034621~cellular macromolecular complex subunit organization	7	10	5	3.5E-03
GO:0006974~response to DNA damage stimulus	7	10	4	4.3E-03
GO:0006461~protein complex assembly	8	11	4	4.6E-03
GO:0070271~protein complex biogenesis	8	11	4	4.6E-03
GO:0030261~chromosome condensation	3	4	28	4.8E-03
GO:0065003~macromolecular complex assembly	9	13	3	5.8E-03
GO:0043933~macromolecular complex subunit organization	9	13	3	8.4E-03
GO:0033554~cellular response to stress	8	11	3	8.5E-03
GO:0006323~DNA packaging	4	6	8	1.3E-02
GO:0012501~programmed cell death	8	11	3	1.3E-02
GO:0051276~chromosome organization	7	10	3	1.5E-02
GO:0007017~microtubule-based process	5	7	5	2.1E-02
GO:0008219~cell death	8	11	3	2.8E-02
GO:0016265~death	8	11	3	2.9E-02
GO:0006281~DNA repair	5	7	4	3.0E-02
GO:0010605~negative regulation of macromolecule metabolic process	8	11	3	3.1E-02
GO:0043065~positive regulation of apoptosis	6	9	3	3.2E-02
GO:0043068~positive regulation of programmed cell death	6	9	3	3.3E-02
GO:0010942~positive regulation of cell death	6	9	3	3.4E-02
GO:0006915~apoptosis	7	10	3	3.7E-02
GO:0006917~induction of apoptosis	5	7	4	4.3E-02
GO:0012502~induction of programmed cell death	5	7	4	4.4E-02
GO:0042981~regulation of apoptosis	8	11	2	4.7E-02
GO:0043067~regulation of programmed cell death	8	11	2	4.9E-02
GO:0010941~regulation of cell death	8	11	2	5.0E-02

Supplemental Table 6. Authentications of human cell lines by short tandem repeat (STR) DNA profiling analysis.

H157		
**Amelogenin**	**X**	**X**
CSF1PO	12	12
D13S317	12	12
D16S539	12	13
D18S51	13	15
D19S433	11	13
D21S11	32	32
D2S1338	21	22
D3S1358	17	18
D5S818	10	13
D7S820	12	12
FGA	22	23
THO1	7	9
TPOX	6	12
vWA	15	15

H1650		
**Amelogenin**	**X**	**X**
CSF1PO	11	11
D13S317	11	11
D16S539	11	12
D18S51	10	10
D19S433	15	15
D21S11	30	30
D2S1338	19	19
D3S1358	18	18
D5S818	11	11
D7S820	8	9
D8S1179	12	12
FGA	20	20
THO1	9.3	9.3
TPOX	11	11
vWA	18	18

H520		
**Amelogenin**	**X**	**X**
CSF1PO	10	10
D13S317	10	11
D16S539	8	13
D18S51	17	17
D19S433	13	14
D21S11	30	30
D2S1338	18	23
D3S1358	16	16
D5S818	12	13
D7S820	8	12
D8S1179	14	15
FGA	22	22
THO1	10	10
TPOX	8	8
vWA	18	19

H3255		
**Amelogenin**	**X**	**X**
CSF1PO	11	14
D13S317	11	13
D16S539	8	8
D18S51	12	12
D19S433	14	15
D21S11	28	28
D2S1338	17	19
D3S1358	14	18
D5S818	11	12
D7S820	10	13
D8S1179	13	13
FGA	23	25
THO1	6	6
TPOX	8	11
vWA	16	16

H460		
**Amelogenin**	**X**	**Y**
CSF1PO	11	12
D13S317	13	13
D16S539	9	9
D18S51	13	15
D19S433	14	14
D21S11	30	30
D2S1338	17	25
D3S1358	15	18
D5S818	9	10
D7S820	9	12
D8S1179	12	12
FGA	21	23
THO1	9.3	9.3
TPOX	8	8
vWA	17	17

Supplemental Figures

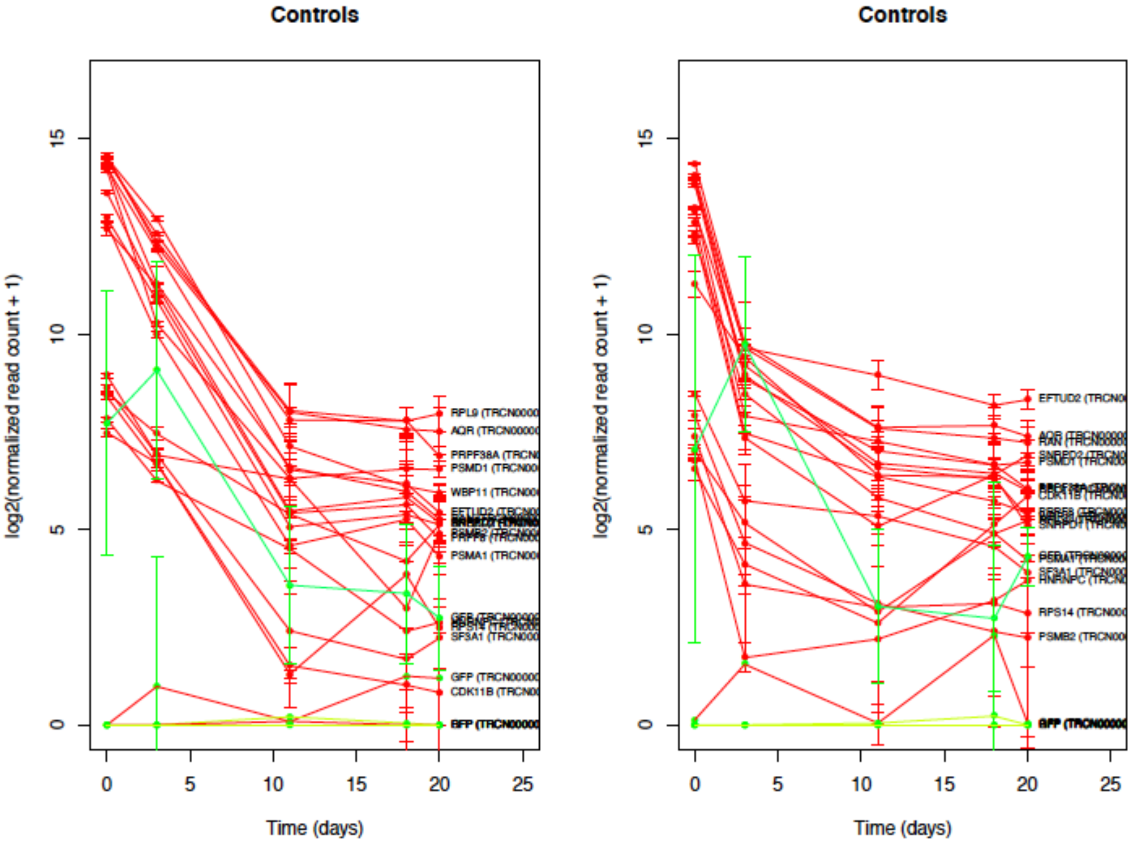

Supplemental Figure 1. Depletion of all 19 control hairpins essential for cell survival over time validates the screen's functionality.

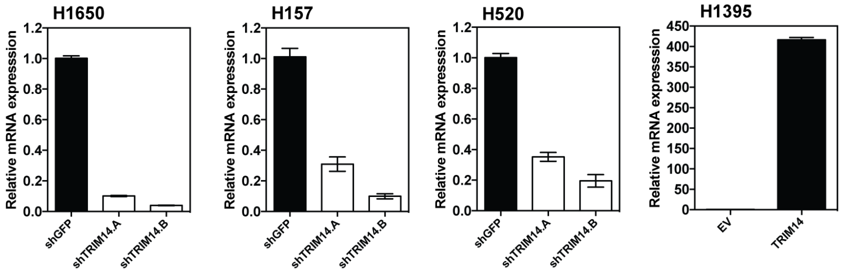

Supplemental Figure 2. Relative mRNA levels of *TRIM14* in isogenic cell lines. RT-qPCR was used to confirm that mRNA levels for *TRIM14* were reduced in shRNA infected cells compared to controls.

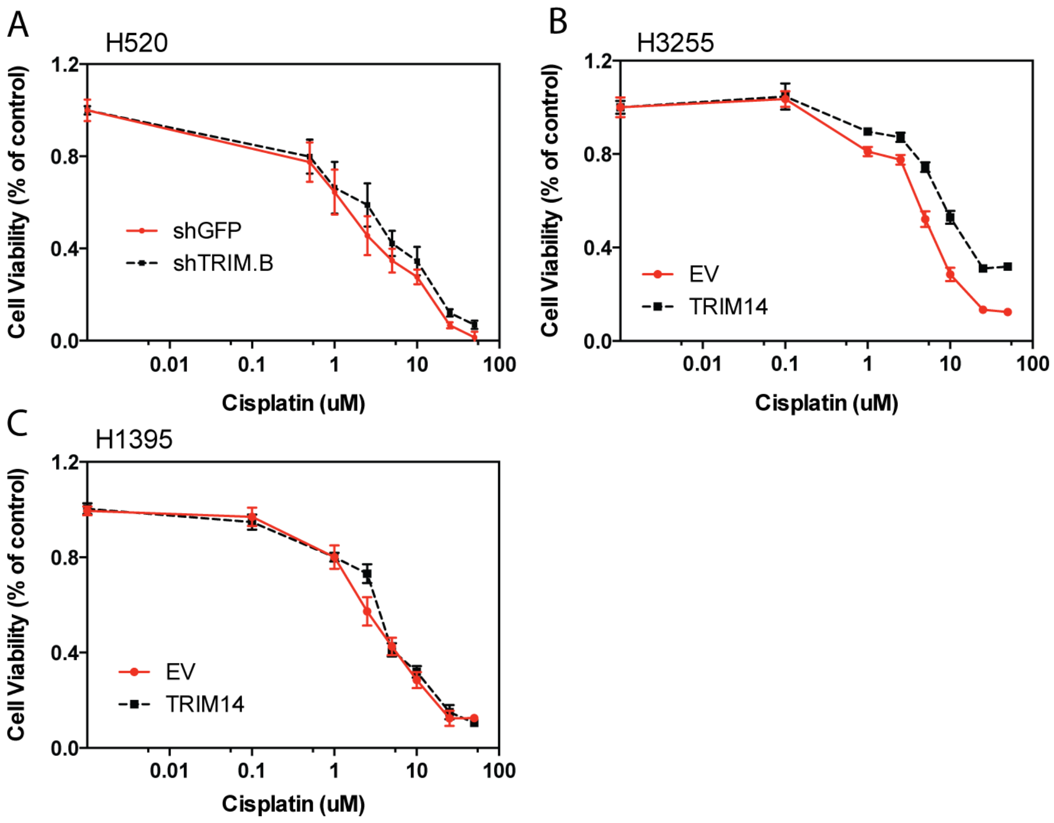

Supplemental Figure 3. TRIM14 expression does not alter the sensitivity of cell lines to cisplatin.

(A-C) NSCLC cell lines were treated with serial dilutions of cisplatin for 48 hours and cell viability was measured by MTS assays. Control cell lines are labeled red. Results shown represent more than three biological replicates.

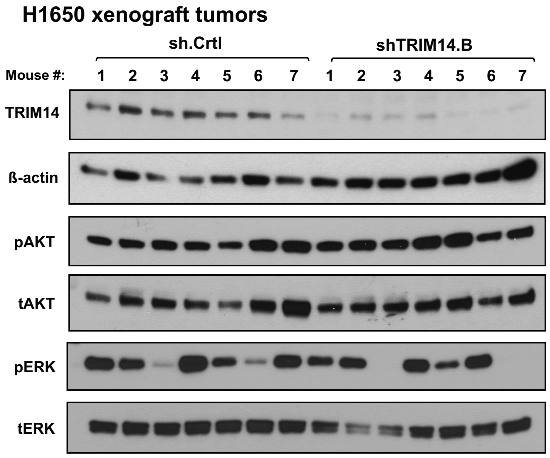

Supplemental Figure 4. TRIM14 suppression in NSCLC cells has no effect on canonical proliferative MAPK or AKT pathways *in vivo*.
Total protein extracts from H1650 xenograft tumours were subjected to Western blot analysis using indicated antibodies and compared with control cell lines. β-actin was used as a loading control.

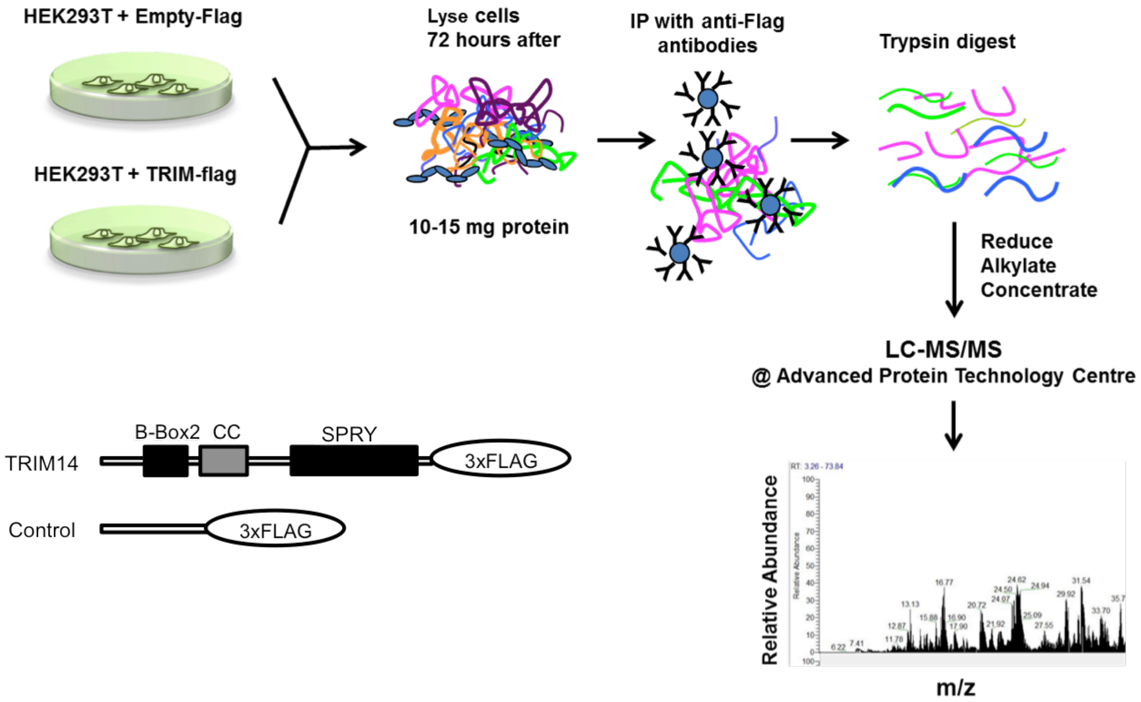

Supplemental Figure 5. Schematic diagram illustrates the experimental design to identify novel binding partners of TRIM14 using mass spectrometry.

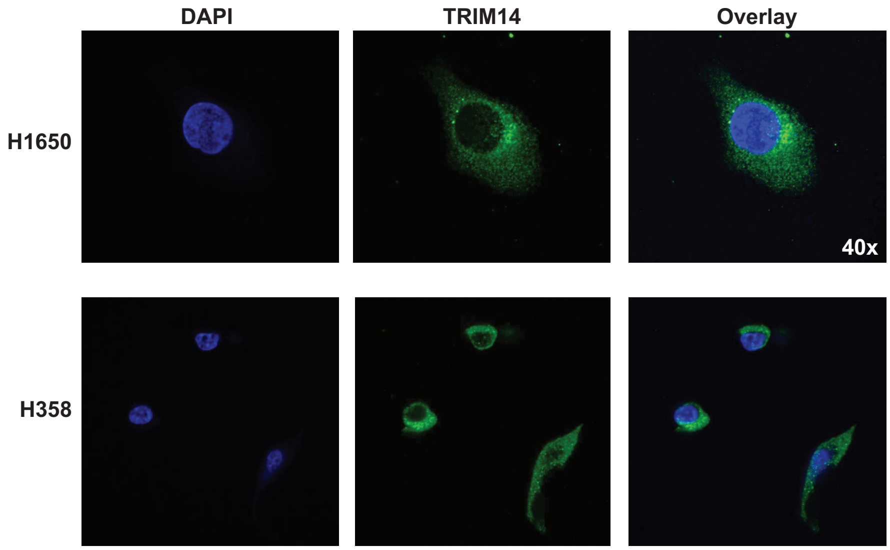

Supplemental Figure 6. TRIM14 is predominantly localized to the cytosol in NSCLC cells. Confocal microscopy visualization of endogenous TRIM14 (green) in H1650 (A) and H358 (B) reveals localization in the cytosol. DAPI (blue) was used to visualize nucleus structures.

Supplemental Figure 6. Uncropped original western blots for Figures 1 and 2.

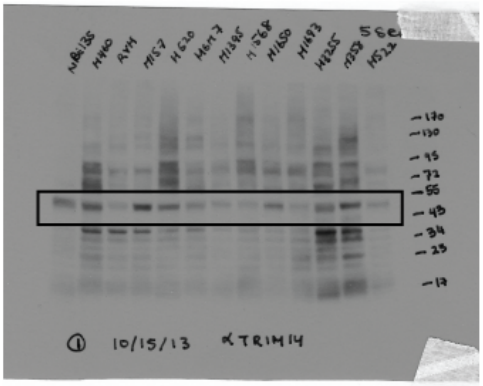

Figure 1b: TRIM14

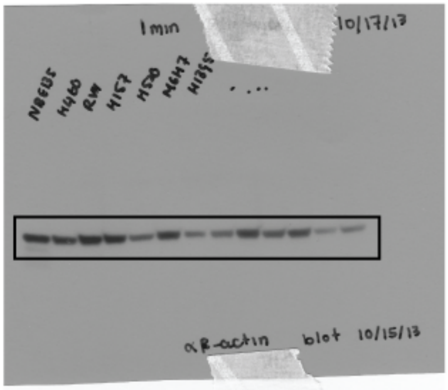

Figure 1b: β-actin

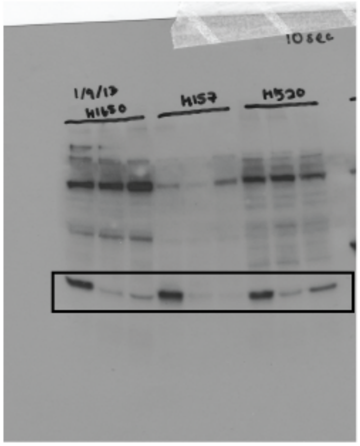

Figure 1c: TRIM14

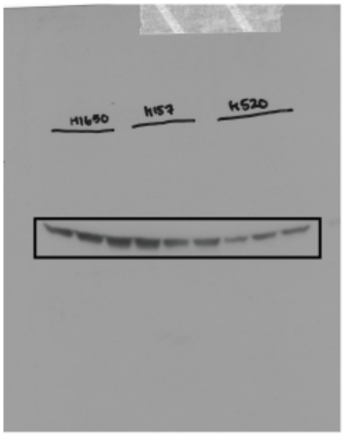

Figure 1c: β-actin

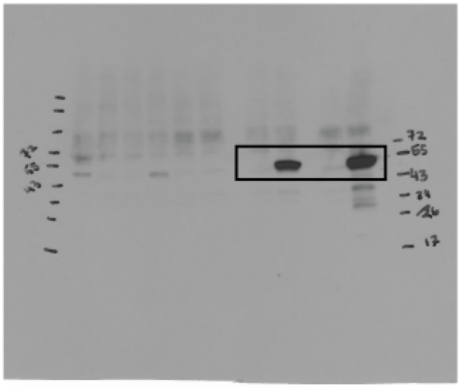

Figure 1c: TRIM14

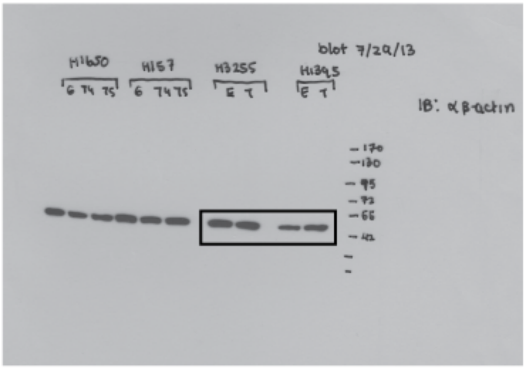

Figure 1c: β-actin

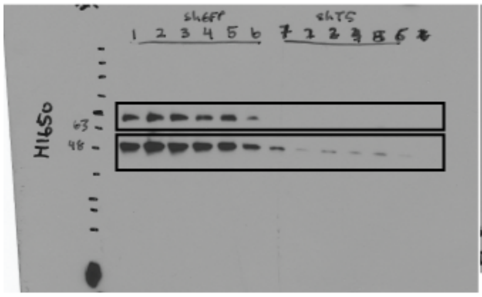

Figure 2c: AIF and TRIM14

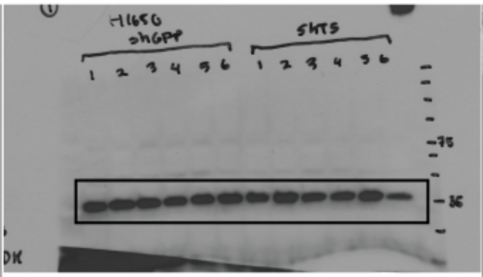

Figure 2c: GAPDH

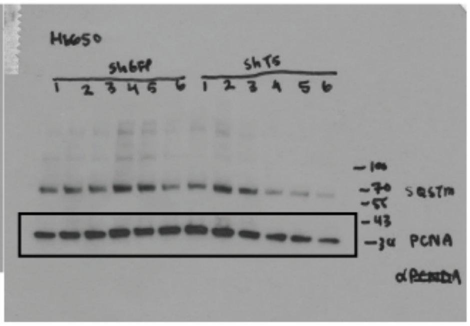

Figure 2c: PCNA

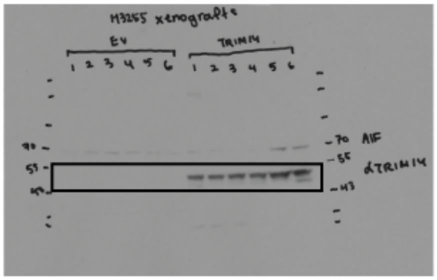

Figure 2c: AIF and TRIM14

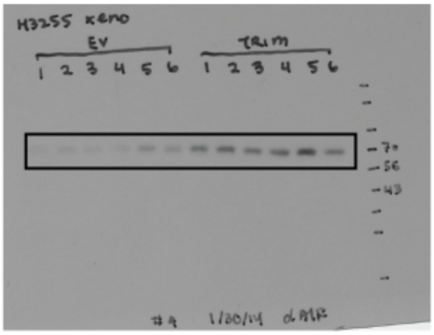

Figure 2c: AIF

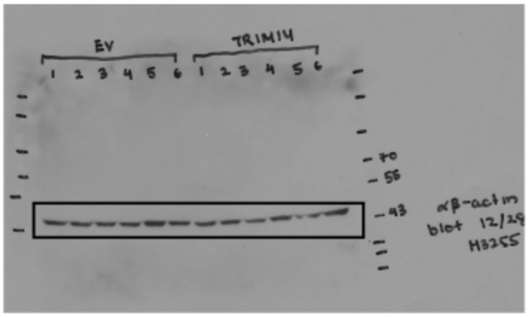

Figure 2c: β-actin

Supplemental Figure 7. Uncropped original western blots for Figures 4 and 5.

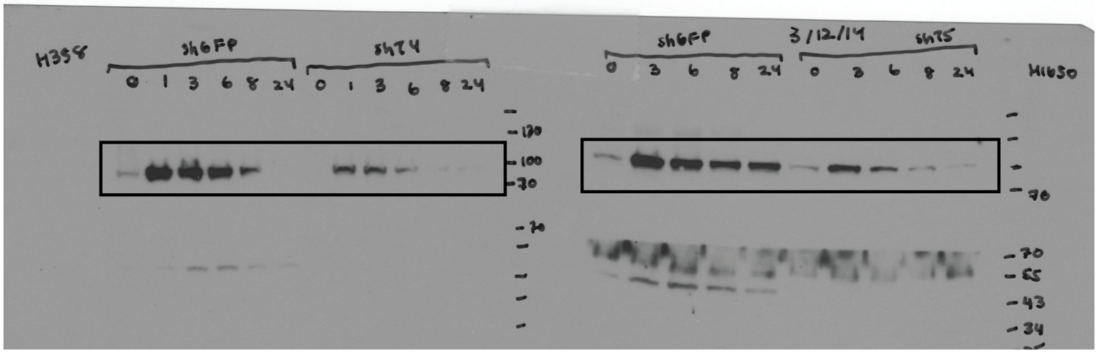

Figure 4E: p-STAT1 Y701

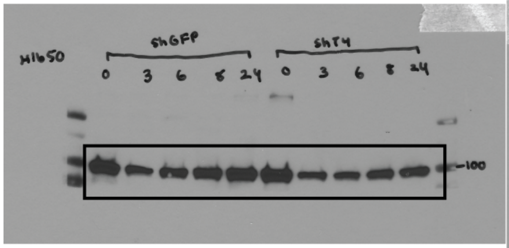

Figure 4E: total STAT1

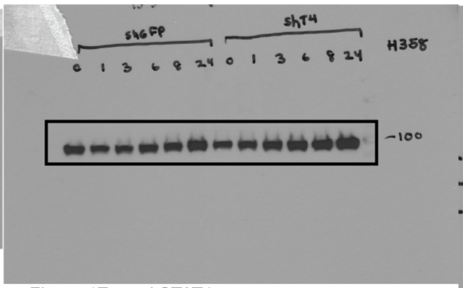

Figure 4E: total STAT1

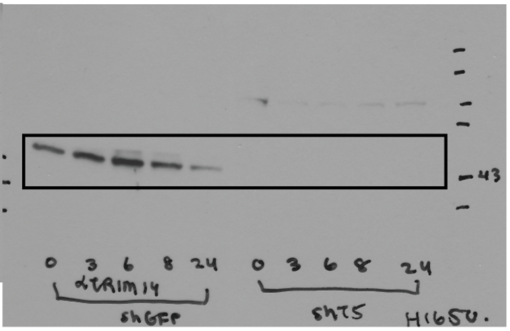

Figure 4E: TRIM14

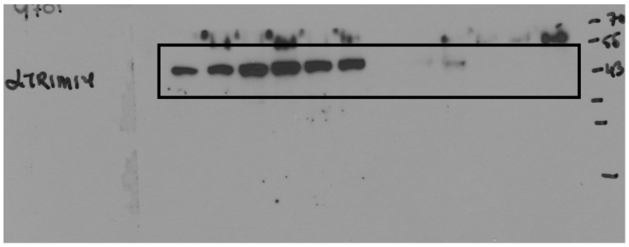

Figure 4E: TRIM14

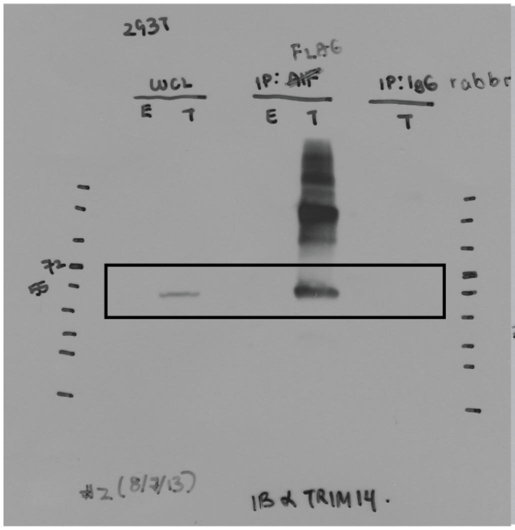

Figure 4E: TRIM14

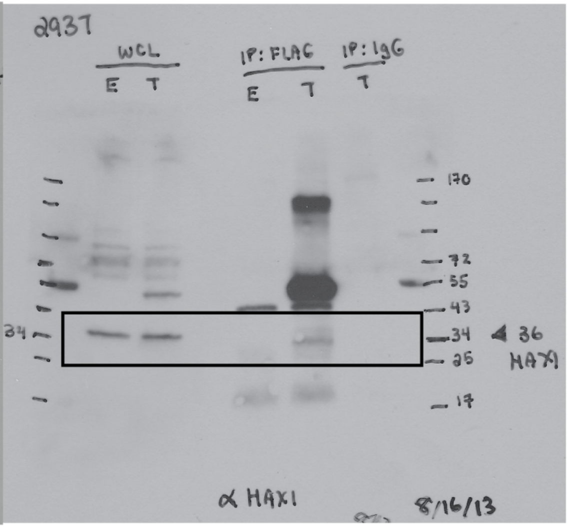

Figure 4E: HAX1

Supplemental Figure 8. Uncropped original western blots for Figure 5.

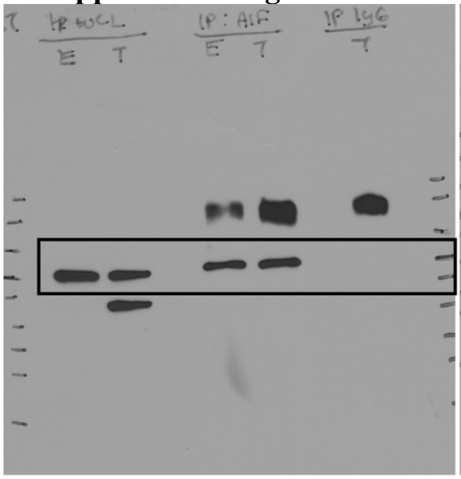

Figure 5B: AIF

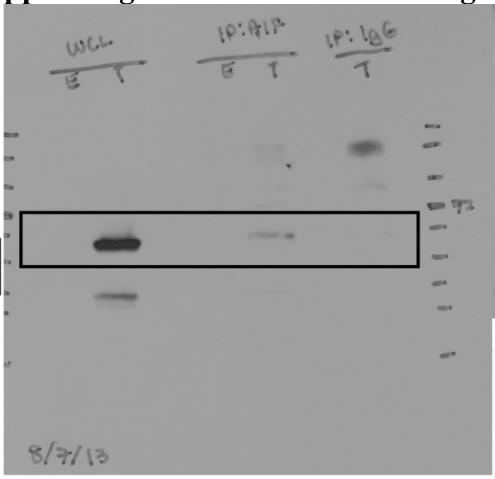

Figure 5B: FLAG

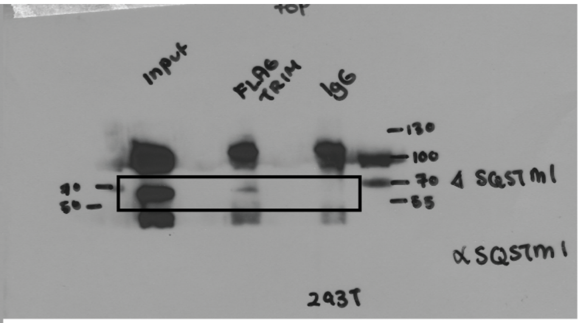

Figure 5C: p62

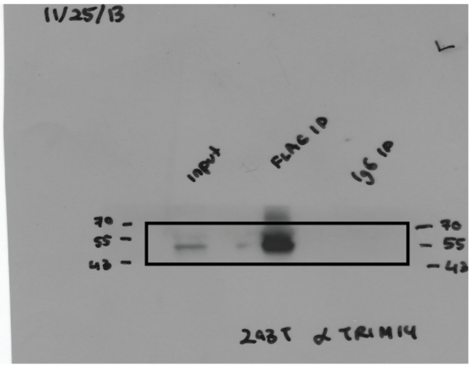

Figure 5C: TRIM14

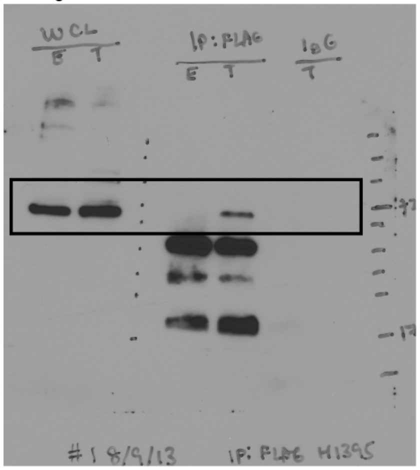

Figure 5D: p62

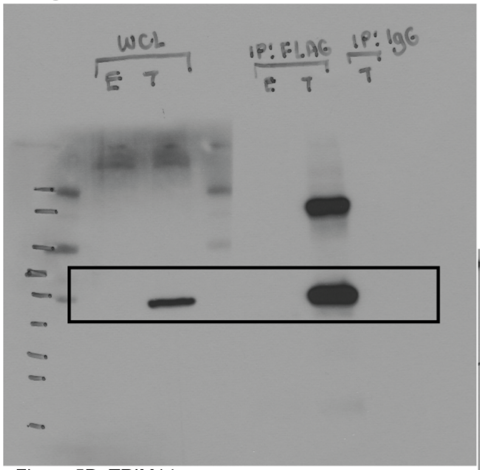

Figure 5D: TRIM14

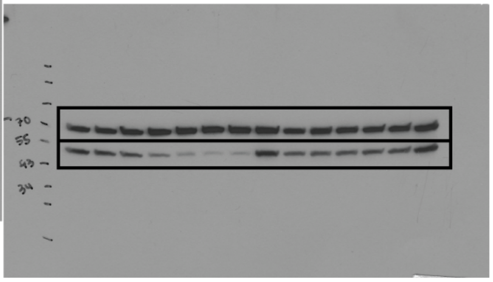

Figure 5D: HSP70 and TRIM14

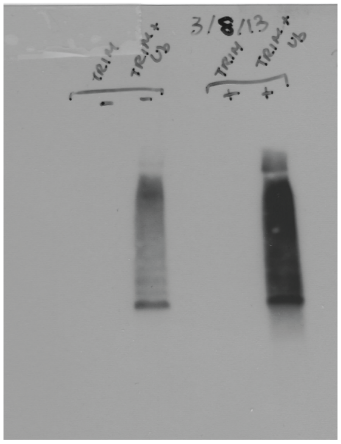

Figure 5F: HA

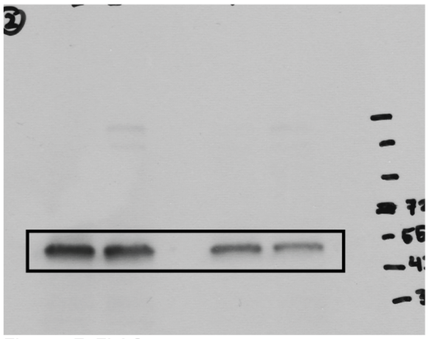

Figure 5F: FLAG

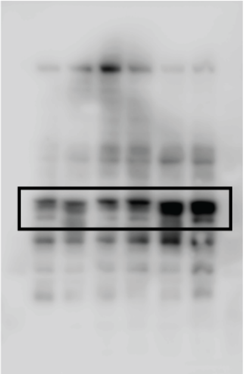

Figure 5H: TRIM14

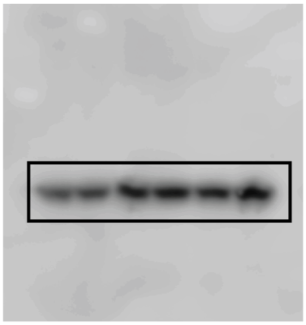

Figure 5H: β-actin

Supplemental References

1 Zhu, C. Q. *et al.* Prognostic and predictive gene signature for adjuvant chemotherapy in resected non-small-cell lung cancer. *J Clin Oncol* **28**, 4417-4424, doi:10.1200/jco.2009.26.4325 (2010).

2 Marcotte, R. *et al.* Essential gene profiles in breast, pancreatic, and ovarian cancer cells. *Cancer discovery* **2**, 172-189, doi:10.1158/2159-8290.cd-11-0224 (2012).

3 Keller, A., Nesvizhskii, A. I., Kolker, E. & Aebersold, R. Empirical statistical model to estimate the accuracy of peptide identifications made by MS/MS and database search. *Anal. Chem.* **74**, 5383-5392 (2002).

4 Winton, T. *et al.* Vinorelbine plus cisplatin vs. observation in resected non-small-cell lung cancer. *N Engl J Med* **352**, 2589-2597, doi:10.1056/NEJMoa043623 (2005).

5 Shedden, K. *et al.* Gene expression-based survival prediction in lung adenocarcinoma: a multi-site, blinded validation study. *Nat Med* **14**, 822-827, doi:10.1038/nm.1790 (2008).

6 Raponi, M. *et al.* Gene expression signatures for predicting prognosis of squamous cell and adenocarcinomas of the lung. *Cancer Res* **66**, 7466-7472, doi:10.1158/0008-5472.can-06-1191 (2006).

7 Der, S. D. *et al.* Validation of a histology-independent prognostic gene signature for early-stage, non-small-cell lung cancer including stage IA patients. *J Thorac Oncol* **9**, 59-64, doi:10.1097/jto.0000000000000042 (2014).
